# Analytical validation of a highly accurate and reliable next-generation sequencing-based urine assay

**DOI:** 10.1128/spectrum.02026-25

**Published:** 2026-04-21

**Authors:** Mara Couto-Rodriguez, David C. Danko, Heather L. Wells, Sol Rey, Xavier Jirau Serrano, Gabor Fidler, John Papciak, P. Ford Combs, Anna Plourde, Michael Augenbraun, Christopher E. Mason, Caitlin Otto, Niamh B. O'Hara, Dorottya Nagy-Szakal

**Affiliations:** 1 Biotia Inc., New York, New York, USA; 2 The Department of Medicine, SUNY Downstate Health Sciences University 12298, New York, New York, USA; 3 Tri-Institutional Computational Biology & Medicine Program, Weill Cornell Medicine of Cornell University, New York, New York, USA; 4 Weill Cornell Medicine, The HRH Prince Alwaleed Bin Talal Bin Abdulaziz Alsaud Institute for Computational Biomedicine, New York, New York, USA; 5 Weill Cornell Medicine, The WorldQuant Initiative for Quantitative Prediction, New York, New York, USA; 6 Weill Cornell Medicine, The Feil Family Brain and Mind Research Institute, New York, New York, USA; 7 The Department Cell Biology/College of Medicine, SUNY Downstate Health Sciences University 12298, New York, New York, USA; University of Delhi, Delhi, India

**Keywords:** clinical metagenomics, next-generation sequencing, urinary tract infection, analytical validation, antimicrobial stewardship, machine learning, infectious disease, urogenital pathogens, clinical diagnostics, precision medicine

## Abstract

Urinary tract infections (UTIs) are diagnosed based on symptoms and confirmed by urine culture, despite its limitations in sensitivity. False-negative cultures can lead to inappropriate antimicrobial use or urosepsis in high-risk patients. Next-generation sequencing (NGS)-based metagenomics offers a comprehensive and precise alternative but is rarely applied clinically. We developed and validated BIOTIA-ID, a clinical-grade NGS-based diagnostic pipeline for pathogen detection in urine. Remnant clinical and spiked urine samples underwent extraction, metagenomic library preparation, and Illumina NextSeq 550 sequencing. We trained and applied a bioinformatic pipeline that uses machine learning to identify pathogens and resistance markers. BIOTIA-DX was intentionally designed and trained to increase stringency and reduce false positive detection of urogenital commensals or opportunistic microbes present at colonization levels. Internal controls ensured standardized, high-stringency results. The assay was validated on 1,470 urine specimens evaluating over 14.5k analytes. The clinical validation achieved a 97.2% sensitivity and 99.6% specificity with a limit of detection (LoD) of <15,000 CFU/mL for most bacterial species and <5,000 CFU/mL for fungal species. Discordant results were reconciled by target-specific qPCR or 16S Sanger sequencing, and 87% of the NGS results were concordant with the comparator. A subset of 332 clinical specimens was tested and validated for antimicrobial resistance (AMR). *sul* and *blaSHV* genes were commonly associated with *Escherichia coli* and *Klebsiella pneumoniae*, while *cfxA* was found in *Prevotella* and *Pseudomonas* spp. detected by BIOTIA-ID. Overall, these data demonstrate that BIOTIA-ID is a comprehensive, highly accurate end-to-end diagnostic assay with notable advantages over current culture-based diagnostics.

**IMPORTANCE:**

Urinary tract infections (UTIs) are among the most common infections, yet current diagnostic methods, including urine culture, often fail to detect pathogens accurately, leading to delayed treatment and inappropriate antimicrobial use. Clinical metagenomics offers a powerful alternative, especially in complicated cases. BIOTIA-ID is a validated, clinical-grade next-generation sequencing (NGS)-based assay that provides highly accurate pathogen identification and antimicrobial resistance profiling. By incorporating machine learning and stringent quality controls, BIOTIA-ID minimizes false positives and enhances diagnostic precision. Our study demonstrates its superior performance over culture, with potential to improve UTI diagnostics, guide targeted therapy, and support antimicrobial stewardship. The implementation of urine metagenomic diagnostics could support recurrent and complicated UTI patient management, providing a more reliable alternative to traditional methods.

## INTRODUCTION

Annually, 11 million people in the United States and 404 million people worldwide are diagnosed with a urinary tract infection (UTI) (1, 2). Immunocompromised patients and patients with urological conditions are at higher risk to develop complicated and/or recurrent UTI (cUTI/rUTI), as well as to progress to urosepsis, resulting in higher morbidity and mortality and increased cost of care (3–6). In hospitalized patients, UTIs are associated with 2.3% of the mortality rate and an estimated annual cost of $340 to $450 million in the United States alone (1). Approximately 30% of patients with sepsis have an infection originating from the urogenital tract, with a multinational study revealing that 12% of nosocomial UTI patients progressed to urosepsis (5, 6). Rapidly identifying UTI pathogens in high-risk patients is crucial to administering an appropriate treatment and minimizing unnecessary broad-spectrum antibiotic use.

Urine culture is the standard of care (SOC) for the detection and identification of pathogens causing UTIs. However, culture-dependent approaches are focused on an Enterobacterales-centric paradigm, which is based on assumptions that urine is sterile (7, 8), disregards mixed infections as contamination (9), and is based on clinical studies employing heterogeneous definitions and thresholds for a UTI (10). Culture has several limitations, including (i) an inability to identify hard-to-grow microbial organisms; (ii) challenges in identifying rare pathogens or organisms not routinely cultured; and (iii) risk of false negative results for patients being treated with antibiotics (11–13). Long diagnostic turnaround time (TAT) or inconclusive results could lead to the empiric treatment of suspected infections with broad-spectrum antibiotics that are often inappropriate and may contribute to increased rates of drug resistance (14).

The emergence of new molecular and culture-based techniques has underscored the drawbacks of traditional culture and challenged our understanding of UTIs, raising questions around what is considered a urogenital pathogen and the role of mixed bacterial communities in UTIs (9, 11, 15–18). Genomics-based assays enable direct specimen analysis without the need for culturing by sequencing genetic material, which is analyzed for the presence of pathogenic organisms through the detection of their DNA. By comparing detected genetic sequences to a comprehensive microbial genome database, genomics-based tests can accurately and rapidly identify pathogens in cUTI samples, which may have atypical pathogens, multiple co-infecting organisms, and complex drug resistance profiles. Concerns have been raised about the sensitivity of molecular approaches to differentiate between opportunistic and commensal microbes detection and differentiation of colonization versus infection (16, 19, 20). However, careful development of genomics-based assays that center on extensive validation leveraging culture, molecular, and bioinformatics techniques can increase the utility of this methodology in routine clinical practice, thus offering a promising supplemental testing method to the current gold standard UTI testing.

Here, we developed, optimized, and clinically validated a next-generation sequencing (NGS)-based urine assay, BIOTIA-ID, that has high diagnostic sensitivity and specificity and provides comprehensive detection of urogenital pathogens within a single test.

## MATERIALS AND METHODS

### Clinical validation strategy

**Fig 1:**
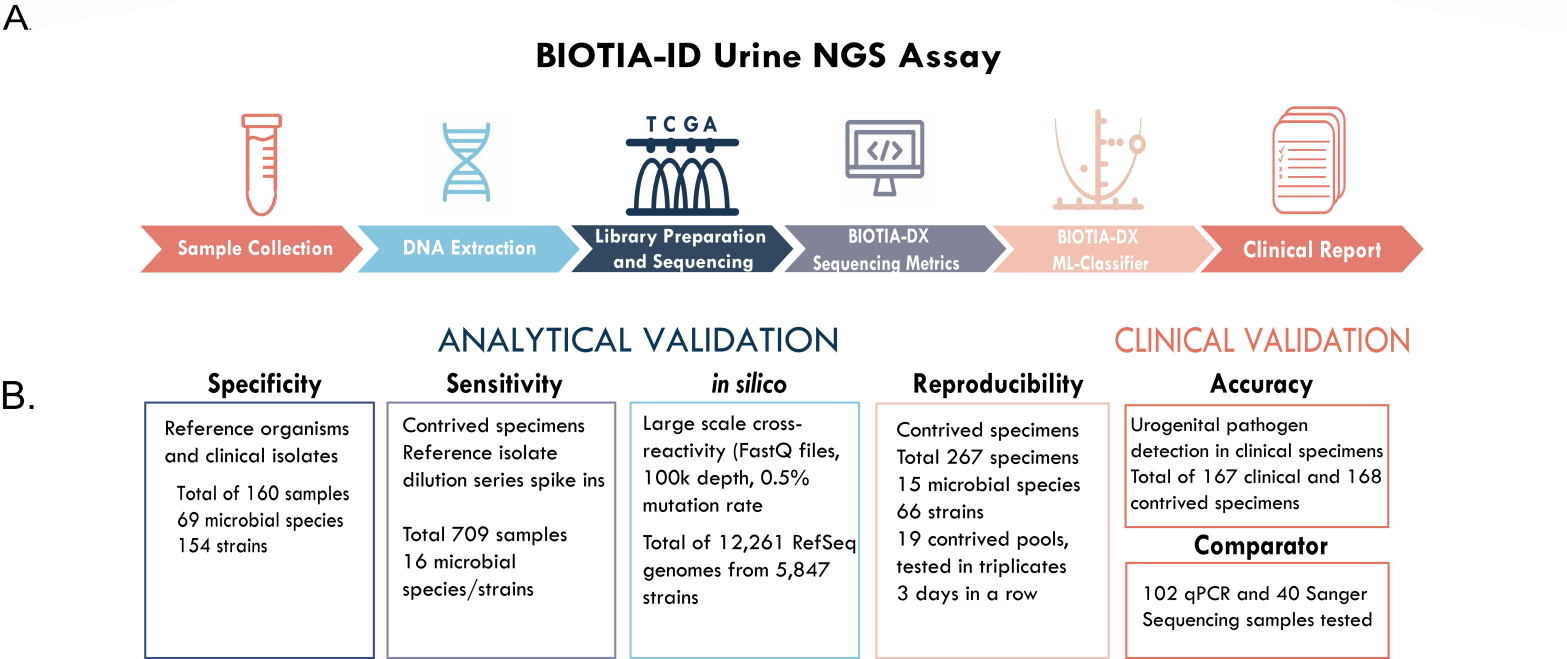
(**A**) An overview of the BIOTIA-ID Urine NGS assay workflow, consisting of sample collection (urine specimens in standard urine transport tubes [UTT]), nucleic acid extraction, library preparation, and sequencing using the Illumina platform, BIOTIA-DX metagenomic analysis, multiple decision tree-based machine learning modeling, and generation of a clinical report with urogenital pathogens detected. (**B**) Analytical validation experiments were conducted with clinical and contrived specimens to assess specificity, sensitivity, and BIOTIA-DX *in silico* performance. The clinical validation established the accuracy of urogenital pathogen detection in clinical specimens with additional comparator studies using qPCR and Sanger sequencing in cases where culture and NGS results disagreed. Furthermore, we completed an inter/intra-assay reproducibility study to show the validity and consistency of our results.

All specimens were processed with our version-controlled BIOTIA-ID Urine NGS assay laboratory protocol, analytical pipeline, and reference microbial database in Biotia’s CLIA-certified laboratory. The assay and clinical validation were designed based on New York State Department of Health validation guidelines for submission of a Laboratory Developed Test for bacteriology and mycology nucleic acid amplification assays (Fig. 1).

### Clinical specimens and reference materials

Deidentified urine specimens in urine transport tubes (UTT) were collected as residual samples after routine clinical testing from different microbiology reference laboratories and were provided with the culture results for each specimen following their standard operating procedures to report pathogens. Samples were collected and processed under an institutional review board (Advarra Pro00038083). A collection of clinical and reference isolates (ATCC, Zeptometrix) was also used for the clinical validation, including bacteria, fungi, viruses, and parasites (listed in Table 1). Reference and clinical microbial isolates used in the contrived specimens were inoculated and grown in Tryptic Soy Agar with 5% Sheep Blood at 37°C for 24–48 h.

**TABLE 1:** Reference organisms and clinical isolates tested and validated on the specificity study^*b*^

Gram-negative enterobacterales	Gram-negative non-enterobacterales	Gram-positive	Fungi
*Citrobacter koseri*	*Acinetobacter calcoaceticus-baumanii-complex*	Other *Staphylococcus* spp.	*Candida albicans*
*Citrobacter freundii*	*Acinetobacter lwolfii*	Anginosus group Streptococci	*Candida auris*
*Enterobacter cloacae-complex*	*Pseudomonas aeruginosa*	*Streptococcus agalactiae*	*Candida dubliniensis*
*Escherichia coli*	*Stenotrophomonas maltophilia*	*Streptococcus mitis*	*Candida glabrata*
*Klebsiella aerogenes*	**Anaerobic bacteria^*c*^**	**Other bacteria**	*Candida guilliermondii*
*Klebsiella oxytoca*	*Anaerococcus* spp.	*Chlamydia trachomatis^a^*	*Candida kefyr*
*Klebsiella pneumoniae-complex*	*Bacteroides fragilis*	*Gardnerella vaginalis*	*Candida krusei*
*Klebsiella variicola*	*Prevotella* spp.	*Mycoplasma genitalium^a^*	*Candida lusitaniae*
*Morganella morganii*	**Gram-positive**	*Mycoplasma hominis^a^*	*Candida parapsilosis*
*Proteus mirabilis*	*Aerococcus* spp.	*Neisseria gonorrhoeae^a^*	*Candida tropicalis*
*Proteus vulgaris*	*Corynebacterium urealyticum*	*Treponema pallidum^a^*	*Cryptococcus neoformans^a^*
*Providencia rettgeri*	*Enterococcus faecalis*	*Ureaplasma* spp*.^a^*	*Rhodoturula mucilaginosa^a^*
*Providencia stuartii*	*Enterococcus faecium*	**Viruses**	*Saccharomyces cerevisiae^a^*
*Raoultella ornithinolytica*	*Staphylococcus aureus*	CMV^*a*^	**Parasites^*a*^**
*Salmonella Typhimurium^a^*	*Staphylococcus epidermidis*	HPV^*a*^	*Cryptosporidium* spp*.^a^*
*Serratia marcescens*	*Staphylococcus lugdunensis*	HSV1^*a*^	*Entamoeba* spp*.^a^*
*Shigella flexneri^a^*	*Staphylococcus saprophyticus*	HSV2^*a*^	*Giardia lamblia^a^*
			*Trichomonas vaginalis^a^*

^*a*^ Organisms tested with BIOTIA-ID assay but not reported as a key urogenital pathogen.

^*b*^ Whole organisms of urogenital pathogens detected by the assay, related organisms, and other organisms that can be present in urine specimens were tested by spiking microbial cells into a negative urine matrix (50,000 CFU/mL for bacteria and 25,000 CFU/mL for fungi). A total of 69 microbial species (bacteria, fungi, viruses, and parasites) and 154 strains were evaluated.

^*c*^ Gray shading and bolded text indicate the headers for the listed pathogens.

### Clinical-grade metagenomic sequencing for infectious disease diagnostics

#### BIOTIA-ID urine NGS assay

Genomic DNA (gDNA) extraction from 2 mL of urine was performed with QIACube-MDx, and yields were quantified with the Qubit Flex. Normalized gDNA was spiked with 5% internal positive control (IPC) and processed with the Illumina DNA prep library preparation kit. Libraries were quality checked for size and concentration with Tapestation 4200 and Qubit Flex, respectively. Libraries were pooled in equimolar concentrations and sequenced on an Illumina NextSeq 550 platform.

#### BIOTIA-DX software

After sequencing, the FastQ files were first subjected to a filtering step, which removed low-quality reads and sequencing adapters. Reads with >40% of bases with phred scores <15, reads with >5 N’s, or reads with complexity <30% were discarded using *fastp* v0.24.0. Detection of the internal positive control was performed for each sample as a confirmation of overall sequencing quality. Human sequence data were then removed from downstream analysis by removing reads which mapped to either the human or chimpanzee genomes. The remaining reads were first compared against a large database of microbial genomes in a coarse classification step using a commonly used metagenomics tool, *kraken2* (21). We use a custom *kraken2* microbial database within BIOTIA-DX that includes curated, high-quality genetic sequences from bacterial, fungal, viral, and non-fungal eukaryotic parasitic organisms. In total, more than 7,000 organisms are represented in the database, with a diverse representation of microbial species and strains.

Despite any degree of database curation, metagenomics tools often result in spurious, false-positive hits, which have historically made it difficult to use NGS in a clinical diagnostic setting. To distinguish between clinically relevant infections, off-target spurious hits by the metagenomics software, or low levels of cross-contamination, we designed a classifier using ML that estimates the probability that a microbial organism is present in a sample. The ML classifier uses a human-validated training set where known pathogens and clinically relevant infections have been verified. These “ground truth” samples were used to train the ML classifier to distinguish between pathogenic infections and non-pathogenic identifications of other organisms. In this way, we can ensure that the ML model is highly tuned to organisms causing clinically significant infections and is not detecting commensal organisms, non-pathogens, or spurious noise in the metagenomics. Microbial targets exceeding predefined *kraken2* thresholds in the coarse classification step are tested using this ML classifier in a fine classification step. Finally, organisms that exceeded a pre-defined probability threshold from the classifier were called present in the sample.

### Analytical validation

#### Specificity

Reference organisms and clinical isolates were tested and validated to establish the specificity of the assay. Whole organisms of urogenital pathogens detected by the assay, genetically related organisms, and other organisms that can be present in urine specimens were tested by spiking microbial cells into a negative urine matrix. A total of 69 microbial species (bacteria, fungi, viruses, and parasites) and 154 strains were evaluated (Table 1). Microbial isolates were spiked into a negative urine matrix at concentrations of 25,000–50,000 CFU/mL and processed with the assay.

#### Analytical sensitivity

The analytical sensitivity was assessed in 16 of the most prevalent urogenital pathogens by spiking reference whole organisms into a negative urine matrix. Serial dilutions of each pathogen were spiked in triplicate, followed by gDNA extraction. The limit of detection was defined as the concentration of the target organism detected in at least 95% of the samples.

### Clinical validation

#### Accuracy

We tested a combination of urine clinical specimens and contrived samples (whole organism microbial reference strains and clinical isolates spiked into negative clinical matrix) to assess accuracy. Deidentified, remnant clinical urine samples were obtained from a reference laboratory and stored at −80°C until processing with the assay. Urine clinical samples were collected based on the pathogens diagnosed by culture. At least 30 specimens, each with the most common urogenital pathogens (*Escherichia coli, Enterococcus faecalis, Klebsiella pneumoniae, Proteus mirabilis,* and *Staphylococcus aureus*), were collected. When fewer than 30 samples for a given pathogen were available, contrived specimens were used to bring the total number of samples for that target to 30 (a total of 335 samples; 167 clinical specimens; and 168 contrived). Contrived samples were used for *S. aureus* (*n* = 30), *P. mirabilis* (*n* = 9), *K. pneumoniae* (*n* = 8), *C. albicans* (*n* = 33), *C. auris* (*n* = 30), *C. glabrata* (*n* = 30), *C. krusei* (*n* = 33), *C. parapsilosis* (*n* = 34), and *C. tropicalis* (*n* = 30), with a spike in concentration close to the limit of detection (LoD) (~2.5–5× LoD). In addition, 35 specimens negative by culture were tested.

See Supplemental materials for additional methodology details on the assay controls, software training set, *in silico* analytical specificity, inter/intra-reproducibility, and comparator testing.

### Antimicrobial resistance gene marker identification

The BIOTIA-DX antimicrobial resistance (AMR) module is designed to detect a comprehensive set of 12 beta-lactamase genes (*blaOXA, blaNDM, blaCTX-M, blaTEM, blaKPC, blaCMY, blaADC, blaSHV, blaVIM, blaPDC, blaZ, cfxA*), four *mec* gene classes (A–D), four *sul* genes (sul1-4), and two *van* genes (vanA-B). Members within these gene families often share a high degree of sequence homology. To address this, gene sequences were aggregated into consensus sequences to enable the general detection of these target gene families. Sequences were collected from the CARD and AMRFinder databases and clustered using CD-HIT with an 80% identity threshold. Gene families with higher levels of sequence diversity (e.g., *blaOXA* or *blaCTX-M*) were represented with multiple clusters covering all variants. The resulting cluster sequences were aligned using MAFFT to construct a base frequency matrix. Consensus sequences for each cluster were defined based on the most frequent nucleotide at each position. This database of gene family consensus sequences was then used as the reference for read mapping in the AMR detection process. We used a subset (*n* = 332) of culture-positive and culture-negative clinical specimens to test for AMR gene markers.

## RESULTS

**Fig 2:**
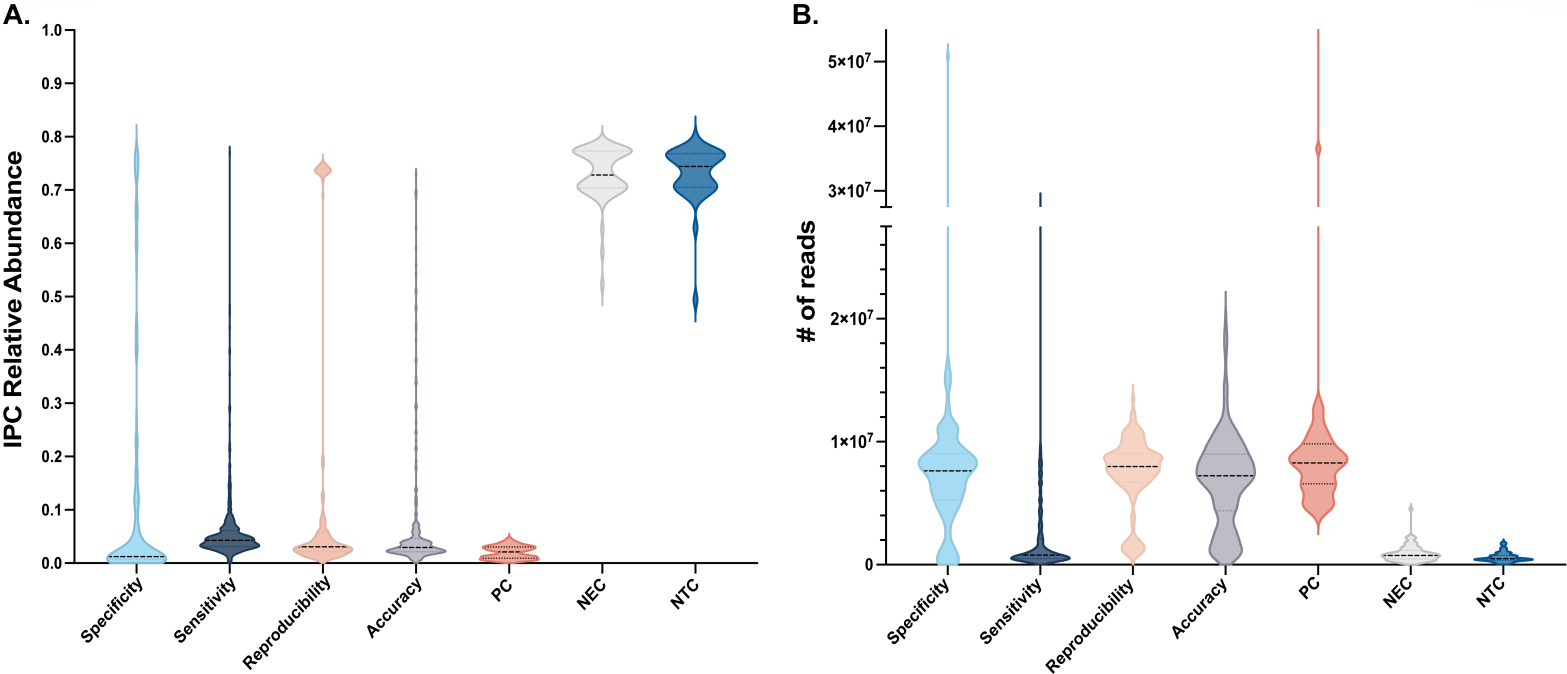
The assay performance of specimens processed in the analytical (specificity, sensitivity, and reproducibility) and clinical validation (accuracy) studies was defined by evaluating the IPC and the number of microbial reads. (**A**) The IPC was detected in all specimens and positive controls (PC) at an expected range (1%–5% abundance). The IPC reads represented the majority of the reads detected in negative extraction controls (NEC) and negative template controls (NTC). (**B**) Violin plots depicting the number of microbial reads obtained in each study and controls. As expected, the clinical and contrived specimens processed through the studies and the PCs generated similar microbial read depth (average 7.9M of microbial reads), while the NEC and NTC had microbial reads below 750k. Violin plots show IPC abundance and microbial read distribution for all samples and controls processed in each study: specificity (light blue, *n* = 160); sensitivity (navy blue, *n* = 709); reproducibility (light pink, *n* = 267); accuracy (gray, *n* = 325), PC (dark pink, *n* = 65), NEC (light gray, *n* = 130), NTC (cerulean blue, *n* = 130).

We developed an end-to-end urine clinical diagnostic metagenomics assay to detect urogenital pathogens and validated its performance on 1,470 samples sequenced in 65 sequencing runs (Fig. 1). The sequencing performance results (microbial read counts and control metrics) are summarized in Fig. 2.

### Key performance characteristics of BIOTIA-ID

#### Specificity

The similarity among different microbial genomes, the diversity of clinical isolates from reference genomes, and contaminating DNA fragments impact the analytical sensitivity of a metagenomic test. Urogenital pathogens detected by the assay and genetically related organisms were processed with BIOTIA-ID (Table 1), yielding a sensitivity and specificity of 100% and 99.96% for bacterial targets and 100% sensitivity and specificity for fungal targets. During assay development, several taxa not extensively represented in the clinical training set generated false positive and false negative results, leading us to process 64 additional species in 80 contrived urine samples to feed more training data to the model. This approach, coupled with hyperparameter tuning of the model, ensured the increased sensitivity and specificity reported above.

#### Sensitivity

**Fig 3:**
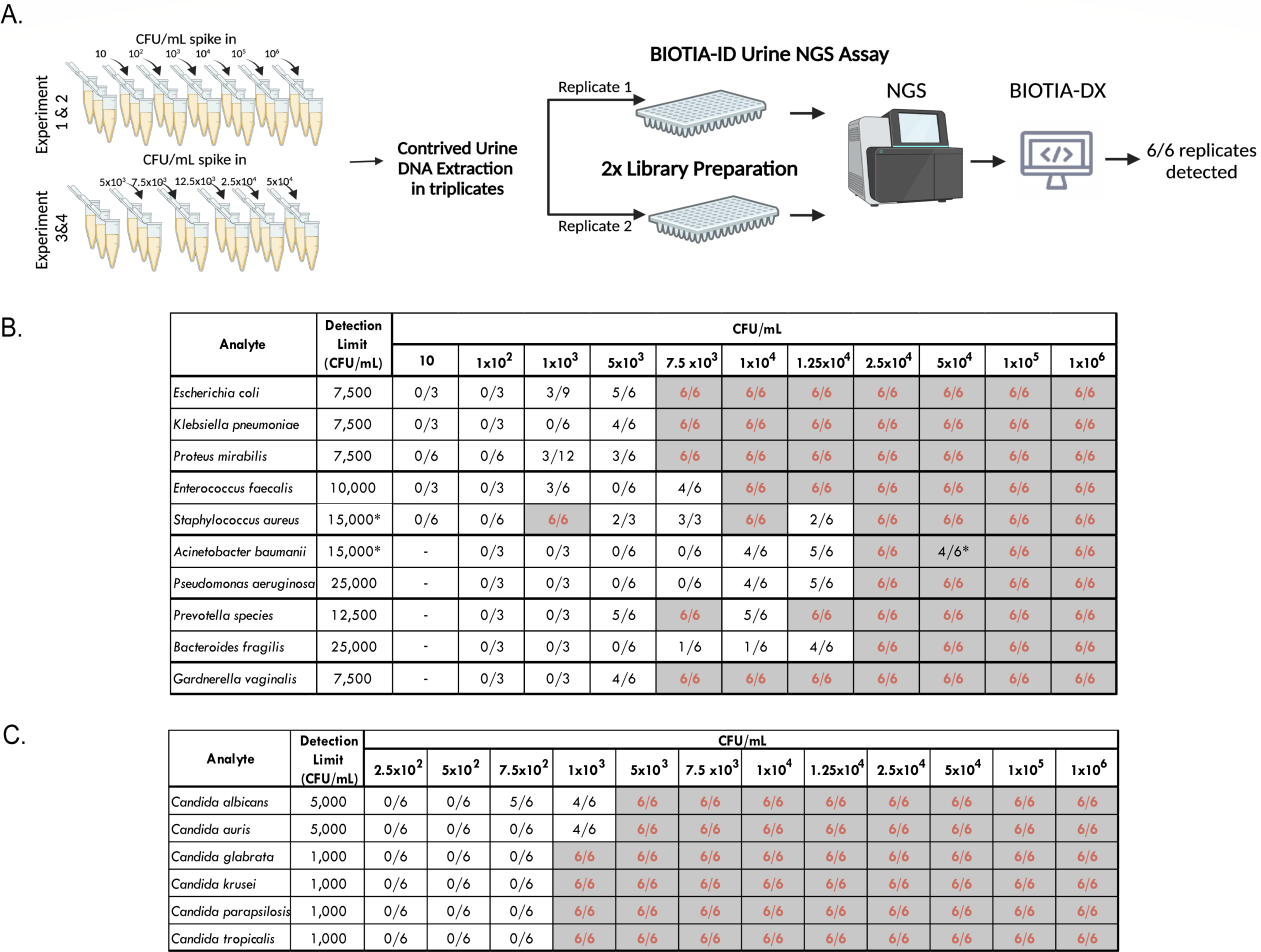
The analytical sensitivity was assessed in the most frequently found urogenital pathogens (10 bacteria and 6 fungal species) by spiking reference whole organisms into a negative urine matrix. (**A**) Each pathogen was spiked in triplicate per concentration (CFU/mL), followed by genomic DNA extraction. Experiments 1 and 2 included six replicates for each concentration of the 10-fold dilution spike-ins, ranging from 10 to 1 million CFU/mL. Experiments 3 and 4 were used to fine-tune the LoD by testing six replicates per concentration, ranging from 5 × 10^3^ to 5 × 10^4^ CFU/mL. (**B and C**) Summary of replicates detected per concentration tested for each urogenital pathogen. The LoD was reproducibly verified and determined based on a 100% positivity rate, resulting in an overall LoD of <25,000 CFU/mL for bacteria and <5,000 CFU/mL for fungi. Specifically, the LoDs determined for each species were 1,000 CFU/mL for *C. glabrata, C. krusei, C. parapsilosis,* and *C. tropicalis*; 5,000 CFU/mL for *C. albicans* and *C. auris*; 7,500 CFU/mL for *E. coli, G. vaginalis, K. pneumoniae,* and *P. mirabilis;* 10,000 CFU/mL for *E. faecalis*; 12,500 CFU/mL for *Prevotella* spp.; 15,000 CFU/mL for *A. baumannii* and *S. aureus*; 25,000 CFU/mL for *P. aeruginosa* and *B. fragilis*.

The analytical sensitivity studies established the lowest concentration of pathogen reliably detected by our assay. Ten bacterial and six fungal species were tested in six replicates per concentration, per analyte evaluated (Fig. 3). A total of 709 contrived specimens were processed. Specifically, the LoDs determined for each species were 1,000 CFU/mL for *C. glabrata, C. krusei, C. parapsilosis,* and *C. tropicalis*; 5,000 CFU/mL for *C. albicans* and *C. auris*; 7,500 CFU/mL for *E. coli, G. vaginalis, K. pneumoniae,* and *P. mirabilis*; 10,000 CFU/mL for *E. faecalis*; 12,500 CFU/mL for *Prevotella* spp.; 15,000 CFU/mL for *A. baumannii* and *S. aureus*; 25,000 CFU/mL for *P. aeruginosa* and *B. fragilis*.

#### Accuracy

The accuracy verification study evaluated the precision of the assay in detecting uropathogens in clinical urine specimens compared to the current gold standard diagnostic (culture). A total of 335 samples (167 clinical and 168 contrived) were tested, including 35 culture-negative specimens.

**Fig 4:**
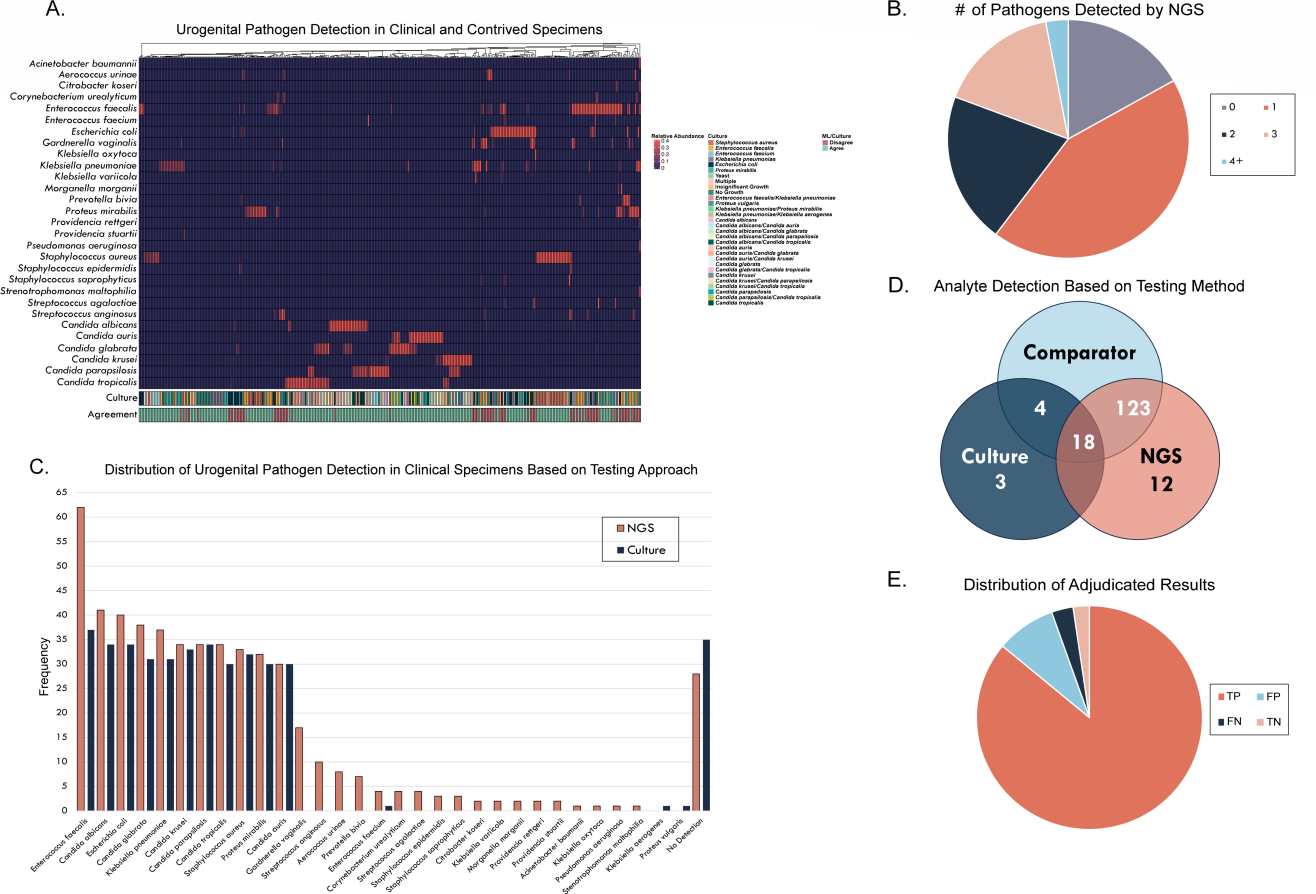
The BIOTIA-ID Urine NGS assay accurately detects and classifies urogenital pathogens in clinical (*n* = 167) and contrived (*n* = 168) specimens in the accuracy study. (**A**) A heatmap depicts the relative abundance of taxa detected by NGS and is annotated with culture diagnosis, and agreement (green) or disagreement (purple) between BIOTIA-DX ML and culture. (**B**) Pie chart showing the distribution of the number of urogenital pathogens detected in clinical urine specimens tested with BIOTIA-ID. (**C**) Bar chart depicts the distribution of microbial analytes detected with culture (navy blue) and with NGS (pink). (**D**) A Venn diagram illustrates the distribution and overlap of the analytes tested and detected with comparator testing for adjudicating the discrepancies found between NGS and SOC. Apparent false positive and false negative results from 19 analytes, where BIOTIA-ID differed from the original culture result (50.2% of clinical samples), were tested by qPCR or Sanger sequencing (*n* = 123), and (**E**) 87% of these NGS results were found to be correct (*n* = 107) as shown on pie chart showing the distribution of adjudicated results. TP: true positive (*n* = 104, dark pink), TN: true negative (*n* = 3, light pink), FP: false positive (*n* = 12, dark blue), FN: false negative (*n* = 4, dark gray). *E. faecalis, K. pneumoniae*, *E. coli, Candida* spp*., Aerococcus* spp*., and G. vaginalis* represented the highest frequency of disagreements (65%) between the SOC and NGS. Comparator reconciled results by taxa tested are shown in Fig. S1. Moreover, analytes adjudicated as false positives by NGS mostly belong to *G. vaginalis* and Anginosus group streptococci, two common opportunistic pathogens of the urogenital tract. However, false negative samples with analytes *E. faecalis, K. pneumoniae,* and *E. coli* yielded below threshold BIOTIA-ID prediction probabilities, demonstrating BIOTIA-DX’s low statistical confidence in defining the presence of the pathogen reported by the SOC.

BIOTIA-ID detected 100% of the spiked microorganisms in contrived specimens and identified a total of 250 urogenital pathogens in the clinical specimens, classifying 83.2% as positive for UTI (Fig. 4A). A single organism was detected in 43.1% of the specimens, while more than one organism was detected in 40.1% (of those 19.8% polymicrobial) (Fig. 4B). Conversely, 72.5% of specimens were observed as single organisms by culture. As depicted in Fig. 4C, BIOTIA-ID detected 26 different urogenital pathogen species with higher frequencies of the key urogenital pathogens used throughout this validation (a total 71.8% of *E. coli, E. faecalis, K. pneumoniae, P. mirabilis,* and *S. aureus*). The remaining 28.2% was uropathogens commonly reportable by culture (e.g., *S. agalactiae*, *Staphylococcus* spp., gram-negative Enterobacterales species, and *Aerococcus* spp.), and organisms not usually culturable with the SOC (e.g., *G. vaginalis* and *Prevotella* spp.). NGS detected *Candida* species in six specimens, while culture reported yeast in only two.

Of the clinical specimens tested, 50.9% revealed a disagreement between NGS and the gold standard culture (11.3% completely disagreed on the species detected). Due to cultural limitations and the high sensitivity of NGS, comparator testing of 123 specimens with 19 analytes (qPCR *n* = 82, Sanger sequencing *n* = 39) was conducted to reconcile discrepancies between findings from both methods. A subset of the 19 concordant analytes was used as clinical positive controls, and 100% of the analytes detected agreed across the three methods (Fig. 4D; Table S5). Apparent false positives and false negatives were then tested, and 87% of these NGS results were concordant with the comparator (Fig. 4E).

Following comparator adjudication, the accuracy study achieved a 97.2% sensitivity and 99.6% specificity (Table 2). Overall, BIOTIA-ID accomplished >99.9% sensitivity and specificity across all samples and analytes processed, exceeding ~14.5k analytes evaluated (Table 2). Contrived specimens yielded >99.9% sensitivity and specificity, and analyte breakdown is presented in Table S6.

**TABLE 2:** BIOTIA-ID clinical and analytical validation performance characteristics summary by analyte category (bacteriology, mycology, overall)^*a*^

Category	Method	TP	TN	FN	FP	Total	Sensitivity (%)	Specificity (%)
Bacteriology	Clinical	210	8,323	7	35	8,575	96.77	99.58
	Contrived	1,099	43,122	0	14	44,048	100.00	99.97
	*In silico*	8,119	272,775	3	147	281,044	99.96	99.95
	Total	9,431	332,483	10	196	341,933	99.89	99.94
Mycology	Clinical	30	530	0	0	560	100.00	100.00
	Contrived	925	9,240	1	2	10,168	99.89	99.98
	*in silico*	4,142	41,420	0	0	45,562	100.00	100.00
	Total	5,097	51,190	1	2	56,290	99.98	100.00
Overall	Clinical	240	8,853	7	35	9,135	97.17	99.61
	Contrived	2,024	52,362	1	16	54,216	99.95	99.97
	*In silico*	12,261	314,195	3	147	326,606	99.98	99.95
	Total	14,528	383,673	11	198	398,223	99.92	99.95

^*a*^ TP, true positive; TN, true negative; FN, false negative; FP, false positive.

### Resistance marker detection using BIOTIA-ID

**Fig 5:**
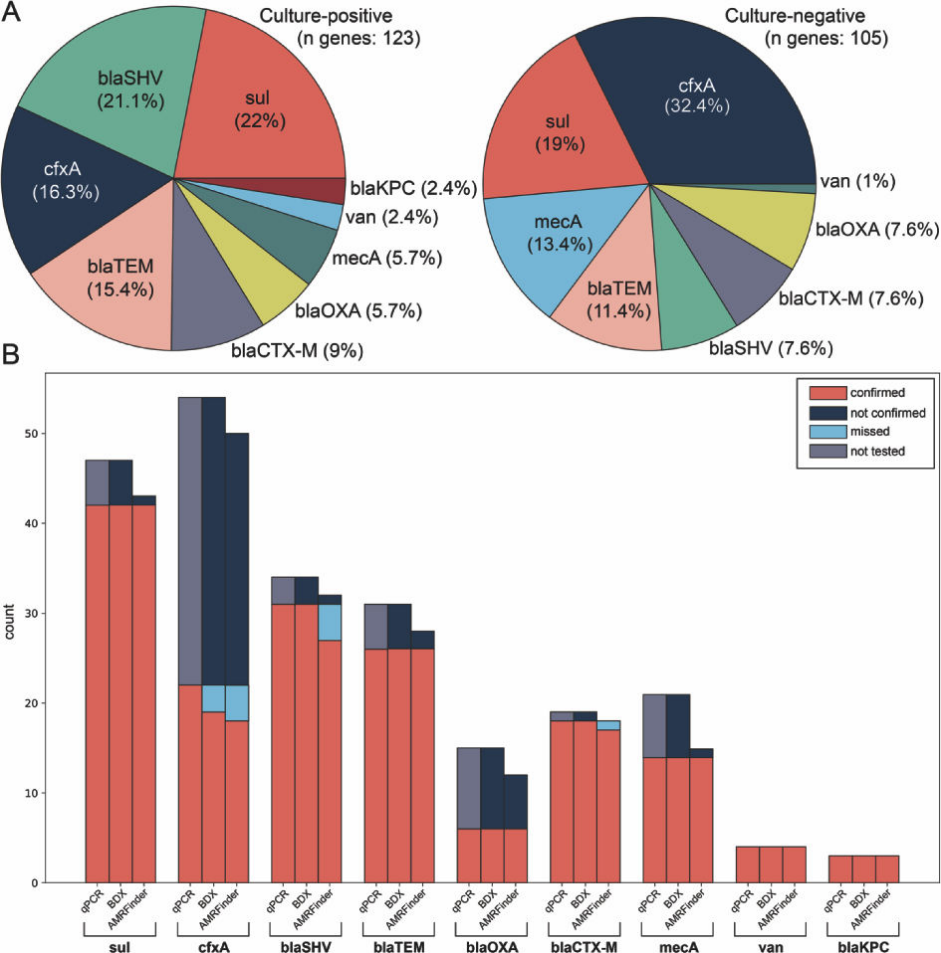
Prevalence of BIOTIA-DX AMR marker detection in both culture-positive and culture-negative clinical specimens where at least one of the target markers was present. (**A**) In culture-positive samples, the most frequently detected markers were *sul, blaSHV,* and *cfxA,* whereas in culture-negative samples, the most prevalent markers were *cfxA, sul,* and *mecA*. Prediction results were compared with AMRFinder, and gene presence was validated with qPCR testing. (**B**) The bar charts display the number of qPCR-confirmed markers (shown in orange). Additionally, the light blue portions represent the false-negative ratios for the two prediction tools. Areas where gene presence was not tested by qPCR are colored in gray, and the dark blue portions indicate the predictions that were not confirmed.

A total of 332 clinical specimens were tested for AMR markers, of which 141 (42%) tested positive for at least one target. The culture-positive (*n* = 131) and culture-negative samples (*n* = 201) exhibited similar proportions of target genes, with 68 and 73 positive markers detected, respectively. Both groups showed comparable AMR profiles, with notable differences in the prevalence of *blaSHV* (21.1% vs 7.6%) and *mecA* (5.7% vs 13.4%) (Fig. 5A). Overall, the most frequently detected markers were *cfxA* (*n* = 54), *sul* (*n* = 47), and *blaSHV* (*n* = 34). The *sul* and *blaSHV* genes were commonly associated with *E. coli* and *K. pneumoniae*, while *cfxA* was found in *Prevotella* and *Pseudomonas* species detected by BIOTIA-ID. The predictions were validated using AMRFinder and confirmed by qPCR testing. Both tools demonstrated 100% accuracy for *sul, blaTEM, blaOXA, mecA, van, and blaKPC*. However, the accuracy for *cfxA* was lower, with BIOTIA-DX achieving 86% and AMRFinder 82%. For three *cfxA* samples incorrectly predicted as negative, PCR confirmation revealed higher cycle threshold (Ct) values (>34) compared to the average Ct of 25 for all *cfxA* samples. Additionally, AMRFinder exhibited reduced accuracy for *blaSHV* (87%) and *blaCTX-M* (94%) compared to BIOTIA-DX (Fig. 5B).

## DISCUSSION

In this study, we developed and validated a clinical-grade NGS assay, BIOTIA-ID, for accurate pathogen identification in urine specimens using ML-classification. BIOTIA-ID overcomes challenges specific to urine molecular diagnostics, including sample collection and transport, low gDNA yields, presence of inhibitors, high human background, and taxonomic bias, due to suboptimal extraction (22–26). The laboratory process is compatible with UTT, the preservative of choice for standard urine cultures in a clinical setting. DNA extraction was optimized for efficient lysis of gram-positive bacteria and fungi, ensuring sufficient gDNA yields from 2 mL of urine, specifically from low biomass samples, such as those collected from males, from catheterized patients, or from patients with prior antibiotic use. Three external controls and one internal control were implemented to validate the process (PC), monitor cross-contamination coming from the process or reagents (negative extraction controls [NEC], negative template controls [NTC]), and ensure the integrity and validity of each sample, minimizing false negatives due to inhibition (IPC). BIOTIA-DX has a clinically curated pathogen database, and the ML-classifier was trained with a combination of urine clinical specimens, clinical and reference isolates, and synthetic metagenomic specimens. Comparator validation of discrepant results, along with a comprehensive training set, increased the specificity and robustness of pathogen prediction. Analytical performance was assessed on 20 prevalent urogenital pathogens in 1,305 contrived samples, 12,250+ *in silico* simulations, and 167 clinical specimens. BIOTIA-ID demonstrated high sensitivity (99.92%) and specificity (99.95%) in predicting uropathogens, making it a promising alternative testing to culture-based diagnostics.

Although urine culture is highly reliable, with reported ~90% sensitivity and 86% specificity in healthy outpatient women, its specificity is exceptionally variable in chronically ill patients (76%–95%) and in patients with indwelling catheters, where it drops close to 0% (27). BIOTIA-ID’s ability to precisely detect pathogens at low concentrations enhances its clinical utility, particularly in the context of managing high-risk immunocompromised patients, rUTI and cUTIs, and/or catheter-associated UTIs. UTI diagnostics currently rely on culture, and while effective, 20%–30% of cases go undiagnosed (13), with this number higher in complicated cases. Culture-based diagnostics fail due to numerous issues, including prior antibiotic exposure, collection or storage errors, cross-contamination, selectivity of culture conditions, and inadequate identification of fastidious, anaerobic, and fungal infections (12, 13), many of which can be addressed with NGS-based diagnostics.

In this study, half of the NGS tested clinical specimens revealed a disagreement with culture findings, with most cases detecting more than one urogenital pathogen, including the species reported by culture. About 11.3% of specimens had different species detected with NGS, and about 15% exhibited clinically relevant discrepancies, like detecting a gram-positive (*E. faecalis*) instead of a gram-negative pathogen (*E. coli*). Despite being highly abundant by both NGS and qPCR testing*, E. faecalis* was the top missed organism by culture, reported as negative. Notably, close to 43% of the clinical specimens which culture diagnosed as *P. mirabilis* showed *E. faecalis* as the dominant pathogen with an NGS-based approach. *E. faecalis* is frequently under-reported by standard culture, with 50% detection rates when compared to Enhanced Quantitative Urine Culture (11). Furthermore, *Candida* species were missed (*n* = 17) or only reported by culture as yeast (*n* = 2), representing 11.9% of the clinical specimens tested. While *Candida* can be isolated with the SOC, sensitivity is rather poor. A study compared *Candida* detection in urine samples with confirmed yeast presence on urinalysis, finding that standard urine culture identified 37% of *Candida* infections, whereas using a fungal-specific media (Sabouraud Dextrose Agar) increased detection to 98% (28). Studies have shown that standard urine culture does not provide optimal growth conditions for all urogenital pathogens, causing organisms, such as Enterobacterales, to outcompete other microbes like *Enterococcus* and *Candida* (11, 15). Failure to detect the correct pathogen results in physicians prescribing inadequate antibiotics, thus exacerbating the emergence of antimicrobial resistance and increasing patient suffering (29, 30).

Like other molecular-based studies, BIOTIA-ID showed improved detection of key urogenital pathogens, atypical microbes, and fastidious bacteria, organisms not reported with culture-based diagnostics (16, 31–34). Although the rise of molecular-based diagnostics and clinical studies using these technologies has revealed that gram-positive, atypical, or fastidious pathogens account for a larger percentage of cUTI infections, more interventional and clinical utilization studies are needed to understand the pathogenesis and epidemiology of these understudied microbes (11, 35). Despite specimen collection bias, BIOTIA-ID identified a significant number of atypical pathogens in the tested clinical specimens, leading us to hypothesize that the detection frequency would likely rise in clinical studies that focus on culture-negative cUTI cases, a population that could greatly benefit from improved diagnostics.

Polymicrobial infections with more than two pathogens are usually considered contamination under the current SOC, and reporting of these results as infection is a topic of debate (10). In accordance with other UTI studies employing molecular approaches, about 40% of clinical specimens tested in this validation showed more than one pathogen detected. Most clinical algorithms designed for uropathogen detection provide optimal growth conditions for a limited number of microbes and are based on a threshold of 10^5^ CFU/mL. Understandably, many guidelines aim to prevent over-prescription of antibiotics. Nonetheless, the development of improved diagnostics can help better guide antimicrobial stewardship efforts while still providing a comprehensive report. Regarding concerns about the potential oversensitivity of an NGS-based assay, BIOTIA-DX was intentionally designed and trained to increase stringency and reduce false positive detection of urogenital commensals or opportunistic microbes present at colonization levels often a weakness of many molecular diagnostic tools and clinical studies (18, 19, 35). Relative abundance of microbial species, percentage of human reads, IPC detection, and complexity of the microbial profiles are features implemented to enhance the BIOTIA-DX’s ability to correctly predict an infection. This study highlights how ML, coupled with extensive laboratory and bioinformatic validation, could improve infectious disease diagnostics by addressing the failures of culture-based diagnostics. Preliminary data suggested a strong linear correlation between spiked CFU/mL and relative abundance of LoD contrived samples (data not shown). The complexity of a clinical urine specimen requires the consideration and development of additional pipeline features for the implementation of a quantification metric that could be relevant and useful to physicians. Additional clinical studies are required to generate a robust data set, facilitating the creation of an updated, user-friendly NGS-based metric that aligns more closely with current clinical and antibiotic stewardship guidelines for UTI management.

Further prospective clinical studies and real-world evaluations are warranted to validate the clinical impact, cost-effectiveness, and health-system benefits of BIOTIA-ID as a UTI diagnostic tool. The observed performance characteristics suggest potential clinical applications where the advantages of accuracy and comprehensive testing outweigh some of the limitations associated with sequencing cost and TAT. Some use cases that would most benefit from this approach include symptomatic patients with negative culture, cUTI and rUTI, and immunocompromised patients with nonspecific infection symptoms who are at high risk of developing sepsis. The current end-to-end turnaround time of approximately 36 h and the expected cost—projected to align with advanced multiplex PCR panels—highlight the emerging feasibility of sequencing-based diagnostics in clinical settings.

BIOTIA-ID represents a significant advancement in UTI diagnostics, offering notable advantages over culture-based methods. Its comprehensive nature, high accuracy, low LoD, and potential to guide targeted antimicrobial therapy support the goals of antimicrobial stewardship. Implementation of BIOTIA-ID in clinical settings with the right diagnostic stewardship has the potential to improve patient outcomes, reduce the misuse of antibiotics, and contribute to the global fight against antimicrobial resistance.

## Data Availability

The data supporting the study findings are available from the corresponding author on request. Sequencing data that support the findings of this study (with human reads removed) have been deposited in GeoSeeq (https://portal.geoseeq.com/projects/bid-urine-analytical-data), a publicly available metagenomic platform, and are available upon request. The BIOTIA-DX software used is described in the Materials and Methods. The software leverages the following openly available tools: fastp v0.23.3, bowtie2 v2.5.1, minimap2 v2.22, SNAP aligner 2.0.3, samtools v1.6, and kraken2 v2.1.3. The ML classifier is a proprietary portion of the code.

## SUPPLEMENTAL MATERIAL

Supplemental material spectrum.02026-25-s0001.pdf Supplemental methods and results; Fig. S1 and S2; Tables S1 to S6.

ASM does not own the copyrights to Supplemental Material that may be linked to, or accessed through, an article. The authors have granted ASM a non-exclusive, world-wide license to publish the Supplemental Material files. Please contact the corresponding author directly for reuse.
