# Supplementary material for: Analytical validation of a highly accurate and reliable next-generation sequencing-based urine assay
Source: Microbiol Spectr. 2026 Apr 21;14(6):e02026-25. doi: 10.1128/spectrum.02026-25 (PMC13227982; doi:10.1128/spectrum.02026-25)
Supplement: Supplemental material — Supplemental methods and results; Fig. S1 and S2; Tables S1 to S6. [file spectrum.02026-25-s0001.pdf]

SUPPLEMENTARY MATERIALS
Supplemental Methods

Assay Controls. Each NGS run includes three external controls and one internal control, which are processed with each batch of samples. The external controls consist of a no template control (NTC, nuclease free water), positive control (PC, Zymobiomics) and negative extraction control (NEC, negative urine matrix). The IPC is a proprietary gDNA that is spiked into each sample at 5%.

Pre-Validation (BIOTIA-DX Training Set). To ensure clinical-grade diagnostic accuracy of these detection thresholds, we trained our ML classifier to recognize clinical-level infections using a training set of clinical samples with known and orthogonally validated infectious organisms. We sequenced and tested a pre-validation sample set of 114 clinical urine samples with known organisms identified by culture, and in cases where our algorithm results differed from culture results, we used PCR testing as a comparator assay. Results from PCR tests were then used to update the list of known organisms in the pre-validation set and re-train the ML classifier with the updated results. The classifier uses a variety of bioinformatic features chosen specifically to distinguish true infections from false positives. These features were calculated for each organism being tested and included statistics such as the depth and evenness of genome coverage, read identity to reference genomes, and the quality of assembled contigs.

In silico Analytical Specificity. To assess the analytical specificity of our pipeline, we used a total of 12,264 RefSeq genomes from 5,847 unique bacteria, eukaryotic organisms, and viruses, to simulate sequencing files with 100,000 reads each. A random mutation rate of 0.5% was applied during simulation to introduce a reasonable degree of variability. Simulated read files were then run through BIOTIA-DX in an identical manner to sequenced laboratory samples and tested for key organisms to assess the potential for organisms outside our laboratory sample set to generate false positive results for key organisms.

Inter/Intra-Reproducibility. For assessment of the reproducibility in bacterial detection, five different pools containing one clinical strain each of *E. coli, E. faecalis, K. pneumoniae, P. mirabilis* and *S. aureus* were tested. Each pool had different strains for each species (altogether 25 different strains) and were spiked into a negative urine matrix at a concentration of 25,000 CFU/mL per analyte. Each pool was then tested daily for three consecutive days in three technical replicates for a total of 15 replicates per pool), 45 replicates per analyte and 6 negative samples. A smaller follow up study sought to evaluate reproducibility of bacterial detection at different concentrations 100,000 CFU/mL (high), 25,000 and 20,000 CFU/mL (medium) and 15,000 CFU/mL (low). Two bacterial pools per concentration were tested in triplicate across three different days for a total of 9 replicates per concentration per analyte. A similar experimental design was performed to assess fungal reproducibility of ten fungal analytes. Five different pools each containing one different strain of *C. albicans*, *C. auris*, *C. glabrata*, *C. krusei*, *C. parapsilosis*, and *C. tropicalis* and a sixth pool with one strain of *C. dubliniensis, C. guilliermondii, C. kefyr* and *C. lusitaniae* were tested in triplicate across three different days. A total of 15 replicates per pool, 45 replicates per analyte and 27 negative samples were evaluated.

Comparator Testing. gDNA from urine specimens was used to perform confirmatory testing of clinical samples yielding results in which NGS and culture findings did not agree. Discrepant results were adjudicated either with qPCR or Sanger Sequencing. Taxa- or group-specific molecular assays were used to amplify analytes of interest (primers and references are listed in **Supplemental Table 1)**. PCR amplicons were sent for Sanger sequencing and consensus sequences were used to determine percent identity of the organism detected in the clinical specimen. A total of 8 different target-specific qPCR assays and 8 Sanger sequencing assays were used to test 123 discrepant taxa and 19 concordant taxa.

Supplemental Results

Inter/Intra-Reproducibility. Qualitative precision was established with inter- and intra-assay reproducibility experiments in which 19 different contrived pools containing at least 3-6 different microbial species were assessed. To account for genetic variation, a total of 66 strains belonging to 15 species of bacteria and fungi were processed. Each pool was tested in triplicate for three consecutive days obtaining a sensitivity, specificity, and qualitative reproducibility of 100%. Detailed results are presented in **Supplemental Table 2**.

In silico Analytical Specificity. The *in silico* studies assessed BIOTIA-DX's ability to identify a large, diverse set of microorganisms absent in clinical or contrived samples and determined whether the pipeline would misidentify closely related organisms (i.e., "cross-reactivity"). The sensitivity and specificity obtained is 99.99% (a detailed breakdown of results is summarized in **Supplemental Table 3)**. Only 1.7% of synthetic samples tested (n=147) showed cross-reactivity and most species observed were within the same genus, do not have clinical relevance, or are unlikely to be found in a urine specimen (**Supplemental Table 4**). Cross-reactivities within the same genus were further differentiated by targeting specific genes containing abundant discriminating sites between species. Cross-reactive samples from 49 species were assigned the correct species in 94% of cases, further reducing false positives to 0.3% (n=27) (**Supplemental Table 4).** BIOTIA-DX overall performance on the training set, specificity, and *in silico* studies is shown on **Supplemental Figure 2**.

Supplemental Figures

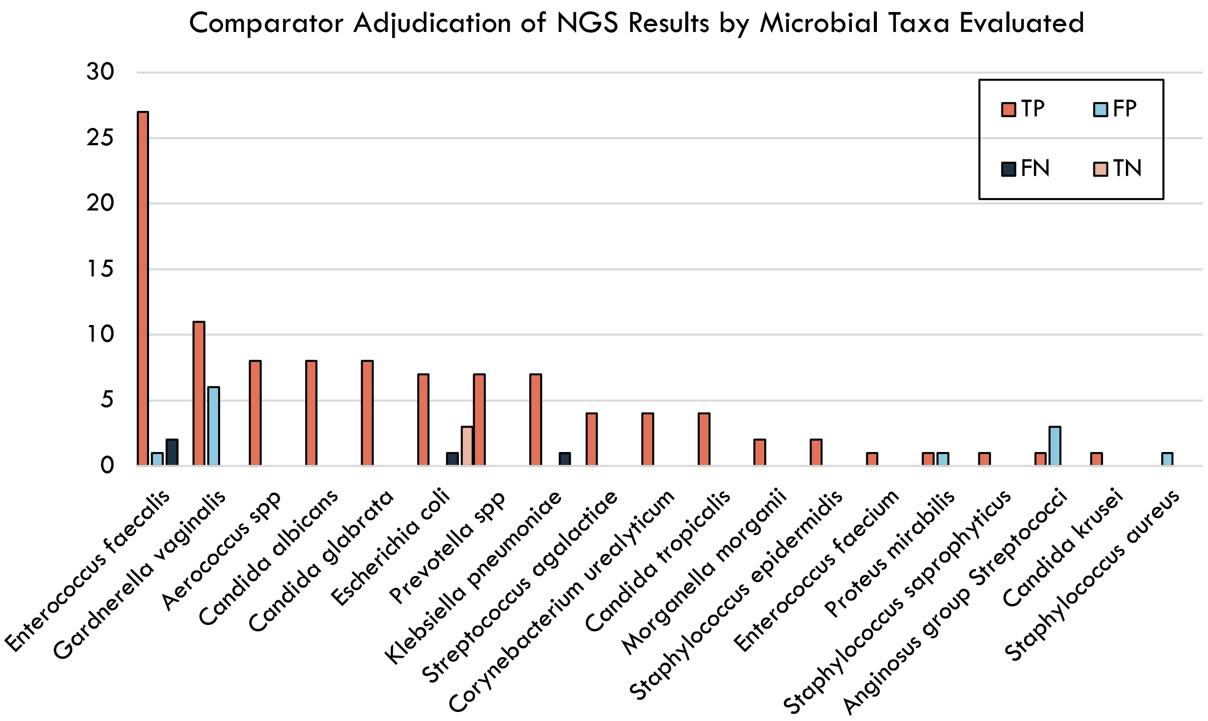

Supplemental Figure 1. Genomic DNA from urine specimens with apparent false positive and false negative results where BIOTIA-ID differed from the original culture result (49.2% of analytes detected), were tested by qPCR or Sanger sequencing (n=123). 87% of NGS results were ultimately found to be correct by the orthogonal adjudication. Bar chart shows comparator adjudicated results for the urogenital pathogens tested with comparator assays. TP: true positive (dark pink), FP: false positive (dark blue), FN: false negative (gray), TN: true negative (light pink).

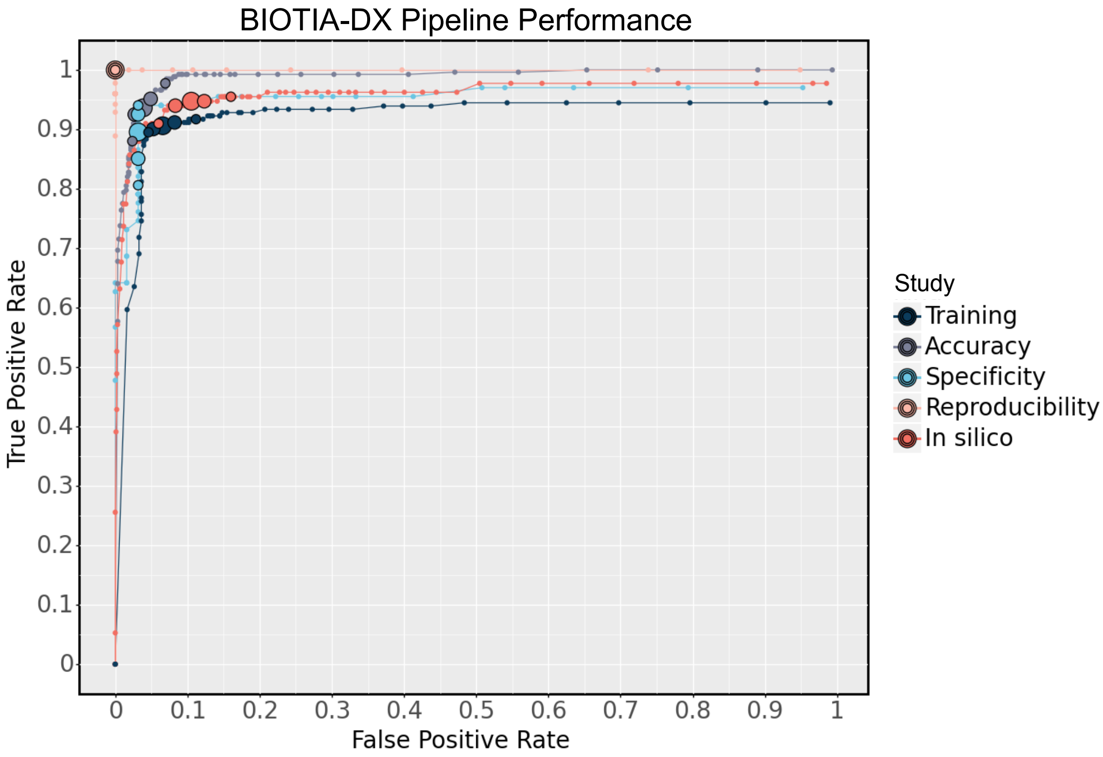

Supplemental Figure 2. BIOTIA-DX ML Performance in samples processed on the training (dark blue), *in silico* (dark pink), accuracy (gray), reproducibility (light pink) and specificity (light blue) studies.

Supplemental Table 1. Summary of comparator assays used in this study

Taxa	Target gene	Assay Type	Forward Primer Sequence	Reverse Primer Sequence	Probe Sequence	Reference
Escherichia coli	uidA	Taqman	CGGAAGCAACGCGTAAACTC	TGAGCGTCGCAGAACATTACA	CGCGTCCGATCACCTGCGTC	Silkie, et al., 2008
Klebsiella pneumoniae	khe	Taqman	GATGAAACGACCTGATTGCATTC	CCGGGCTGTCGGGATAAG	GCGAACTGGAAGGGCCCG	Hartman, et al., 2009
Proteus mirabilis	ureR	Taqman	CCATCAGATTATGTCATTCAA	GAGGAAAATGCAATTTATCTTTA	CACACCCTACCCAACATTCATTTCA	Liu, et al., 2019
Enterococcus faecalis	16S	Taqman	CGCTTCTTTCCTCCCGAGT	GCCATGCGGCATAAACTG	CAATTGGAAAGAGGAGTGGCGGACG	Santo Domingo et al., 2003
Staphylococcus aureus	ebpS	Taqman	CCACATGCCTCTAATAATG	GCGATTTTATTTTCTTTTGTAC	ATGCCATGCCTCCAAATATCGC	Liu, et al., 2019
Gardnerella vaginalis	cpn60	Taqman	CGCATCTGCTAAGGATGTTG	CAGCAATCTTTTCGCCAACT	TGCAACTATTTCTGCAGCAGATCC	Menard et al., 2008
Aerococcus urinae	16S	Taqman	GACGGCTTTGCTGTCACTTATCG	GCTATGCATCATTGSCTTGGTAG	TGCATTAGCTCGTTGGTGGG	Biotia
Prevotella species	16S	SYBR green	GGTTCTGAGAGGAAGGTCCCC	TCCTGCACGCTACTTGGCTG	NA	Stevenson and Weimer, 2007
Candida species	ITS	Sanger	TCCGTAGGTGAACCTGCGG	TCCTCCGCTTATTGATATGC	NA	White et al., 1990
Anaerococcus vaginalis	16S	Sanger	TGATTTCTTCGGAATGAAATTAAGTGGATTA	GGTCATTTTATCATGCGATATTTTGACTTT	NA	Biotia
Corynebacterium urealyticum	16S	Sanger	CCTGCTTGCAGGGTACTCGA	CACCAACCACACTAAAGATTGGTC	NA	Biotia
Enterococcus faecium	16S	Sanger	TTCTTTTTCCACCGGAGCTT	AACCATGCGGTTTYGATTG	NA	Ryu et al., 2013
Morganella morganii	16S	Sanger	AGCTTGCTTCTCTGCTGACGAG	GAGGCCCGAAGGTCCCCCG	NA	Biotia
Staphylococcus epidermidis	16S	Sanger	GTTCAATAGTGAAAGACGGTTTTGCTGTC	GTTTTACGATCCGAAGACCTTCATCAC	NA	Biotia
Staphylococcus saprophyticus	16S	Sanger	TAAAGTGAAAGATGGTTTTGCTATC	GTTTTACGAGCCGAAACCCTTCATCAC	NA	Biotia
Streptococcus agalactiae	16S	Sanger	KGTTTGGTGTTTACACTAGACTG	TTACCGTCACTTGGTAGATTTTCCA	NA	Biotia
Streptococcus anginosus	16S	Sanger	CGTAGTTTACTACACCGTATTCTGTGA	GTACCGTCACTGTGTGAAC	NA	Biotia

Supplementary Table 2A. Summary of Replicates Detected in the Reproducibility Studies

Microbial Species	Replicates Detected	Qualitative Reproducibility
Enterococcus faecalis	81/81	100%
Escherichia coli	81/81	100%
Klebsiella pneumoniae	81/81	100%
Proteus mirabilis	81/81	100%
Staphylococcus aureus	81/81	100%
Candida albicans	45/45	100%
Candida auris	45/45	100%
Candida glabrata	45/45	100%
Candida krusei	45/45	100%
Candida parapsilosis	45/45	100%
Candida tropicalis	45/45	100%
Candida dubliniensis	9/9	100%
Candida guillermondii	9/9	100%
Candida kefyr	9/9	100%
Candida lusitaniae	9/9	100%
Negative	42/42	100%

Supplementary Table 2B. Reproducibility Studies Results by Concentration

Analyte	Strain	Load	Day 1	Day 2	Day 3	Total
Enterococcus faecalis	URV-032-2	15,000	3/3	3/3	3/3	9/9
Escherichia coli	URV-012	15,000	3/3	3/3	3/3	9/9
Klebsiella pneumoniae	URV-155-1	15,000	3/3	3/3	3/3	9/9
Proteus mirabilis	URV-023	15,000	3/3	3/3	3/3	9/9
Staphylococcus aureus	ATCC-43300	15,000	3/3	3/3	3/3	9/9
Enterococcus faecalis	URV-032-2	20,000	3/3	3/3	3/3	9/9
Escherichia coli	URV-012	20,000	3/3	3/3	3/3	9/9
Klebsiella pneumoniae	URV-155-1	20,000	3/3	3/3	3/3	9/9
Proteus mirabilis	URV-023	20,000	3/3	3/3	3/3	9/9
Staphylococcus aureus	ATCC-43300	20,000	3/3	3/3	3/3	9/9
Enterococcus faecalis	URV-032-2	25,000	3/3	3/3	3/3	9/9
Escherichia coli	URV-012	25,000	3/3	3/3	3/3	9/9
Klebsiella pneumoniae	URV-155-1	25,000	3/3	3/3	3/3	9/9
Proteus mirabilis	URV-023	25,000	3/3	3/3	3/3	9/9
Staphylococcus aureus	ATCC-43300	25,000	3/3	3/3	3/3	9/9
Enterococcus faecalis	URV-220	25,000	3/3	3/3	3/3	9/9
Escherichia coli	URV-005-1	25,000	3/3	3/3	3/3	9/9
Klebsiella pneumoniae	URV-038	25,000	3/3	3/3	3/3	9/9
Proteus mirabilis	URV-010	25,000	3/3	3/3	3/3	9/9
Staphylococcus aureus	SJF-038	25,000	3/3	3/3	3/3	9/9
Enterococcus faecalis	URV-032-2	25,000	3/3	3/3	3/3	9/9
Escherichia coli	URV-012	25,000	3/3	3/3	3/3	9/9
Klebsiella pneumoniae	URV-036	25,000	3/3	3/3	3/3	9/9
Proteus mirabilis	URV-014	25,000	3/3	3/3	3/3	9/9
Staphylococcus aureus	SJF-044	25,000	3/3	3/3	3/3	9/9
Enterococcus faecalis	URV-007	25,000	3/3	3/3	3/3	9/9
Escherichia coli	URV-029	25,000	3/3	3/3	3/3	9/9
Klebsiella pneumoniae	URV-022	25,000	3/3	3/3	3/3	9/9
Proteus mirabilis	URV-040	25,000	3/3	3/3	3/3	9/9
Staphylococcus aureus	SJF-034	25,000	3/3	3/3	3/3	9/9
Enterococcus faecalis	URV-026	25,000	3/3	3/3	3/3	9/9
Escherichia coli	URV-006	25,000	3/3	3/3	3/3	9/9
Klebsiella pneumoniae	URV-031	25,000	3/3	3/3	3/3	9/9
Proteus mirabilis	URV-060	25,000	3/3	3/3	3/3	9/9
Staphylococcus aureus	SJF-035	25,000	3/3	3/3	3/3	9/9
Enterococcus faecalis	URV-261	25,000	3/3	3/3	3/3	9/9
Escherichia coli	URV-021	25,000	3/3	3/3	3/3	9/9
Klebsiella pneumoniae	URV-155-1	25,000	3/3	3/3	3/3	9/9
Proteus mirabilis	URV-016	25,000	3/3	3/3	3/3	9/9
Staphylococcus aureus	SJF-041	25,000	3/3	3/3	3/3	9/9
Enterococcus faecalis	URV-032-2	100,000	3/3	3/3	3/3	9/9
Escherichia coli	AR-104	100,000	3/3	3/3	3/3	9/9
Klebsiella pneumoniae	AR-557	100,000	3/3	3/3	3/3	9/9
Proteus mirabilis	AR-156	100,000	3/3	3/3	3/3	9/9
Staphylococcus aureus	SJF-035	100,000	3/3	3/3	3/3	9/9
Candida albicans	60193	25,000	3/3	3/3	3/3	9/9
Candida auris	MYA-5000	25,000	3/3	3/3	3/3	9/9
Candida glabrata	15126	25,000	3/3	3/3	3/3	9/9
Candida krusei	14243	25,000	3/3	3/3	3/3	9/9
Candida parapsilosis	22019	25,000	3/3	3/3	3/3	9/9
Candida tropicalis	1369	25,000	3/3	3/3	3/3	9/9

Candida albicans	10231	25,000	3/3	3/3	3/3	9/9
Candida auris	MYA-5001	25,000	3/3	3/3	3/3	9/9
Candida glabrata	2001	25,000	3/3	3/3	3/3	9/9
Candida krusei	34135	25,000	3/3	3/3	3/3	9/9
Candida parapsilosis	90018	25,000	3/3	3/3	3/3	9/9
Candida tropicalis	1369	25,000	3/3	3/3	3/3	9/9
Candida albicans	14053	25,000	3/3	3/3	3/3	9/9
Candida auris	MYA-5002	25,000	3/3	3/3	3/3	9/9
Candida glabrata	66032	25,000	3/3	3/3	3/3	9/9
Candida krusei	90878	25,000	3/3	3/3	3/3	9/9
Candida parapsilosis	SJF-019	25,000	3/3	3/3	3/3	9/9
Candida tropicalis	750	25,000	3/3	3/3	3/3	9/9
Candida albicans	90029	25,000	3/3	3/3	3/3	9/9
Candida auris	MYA-5003	25,000	3/3	3/3	3/3	9/9
Candida glabrata	90030	25,000	3/3	3/3	3/3	9/9
Candida krusei	6258	25,000	3/3	3/3	3/3	9/9
Candida parapsilosis	SJF-032	25,000	3/3	3/3	3/3	9/9
Candida tropicalis	13803	25,000	3/3	3/3	3/3	9/9
Candida albicans	URV-018	25,000	3/3	3/3	3/3	9/9
Candida auris	CDC B11903	25,000	3/3	3/3	3/3	9/9
Candida glabrata	MYA-2950	25,000	3/3	3/3	3/3	9/9
Candida krusei	URV-019	25,000	3/3	3/3	3/3	9/9
Candida parapsilosis	SJF-033	25,000	3/3	3/3	3/3	9/9
Candida tropicalis	66029	25,000	3/3	3/3	3/3	9/9
Candida dubliniensis	3949	25,000	3/3	3/3	3/3	9/9
Candida guillermondii	6260	25,000	3/3	3/3	3/3	9/9
Candida kefyr	66028	25,000	3/3	3/3	3/3	9/9
Candida lusitaniae	34449	25,000	3/3	3/3	3/3	9/9
Negative Samples	-	-	14/14	14/14	14/14	42/42

Supplementary Table 3A. Performance Characteristics of in silico Studies per Key Urogenital Taxa

Key pathogen	Method	TP	TN	FN	FP	Total	Sensitivity	Specificity
Escherichia coli	Clinical							
	Contrived							
	in silico	3	8239	0	24	8266	100.0%	99.7%
	Total							
Klebsiella pneumoniae	Clinical							
	Contrived							
	in silico	3	8250	0	13	8266	100.0%	99.8%
	Total							
Proteus mirabilis	Clinical							
	Contrived							
	in silico	3	8255	0	8	8266	100.0%	99.9%
	Total							
Enterococcus faecalis	Clinical							
	Contrived							
	in silico	3	8263	0	0	8266	100.0%	100.0%
	Total							
Staphylococcus aureus	Clinical							
	Contrived							
	in silico	3	8252	0	11	8266	100.0%	99.9%
	Total							
Citrobacter species	Clinical							
	Contrived							
	in silico	20	8246	0	0	8266	100.0%	100.0%
	Total							
Enterobacter aerogenes	Clinical							
	Contrived							
	in silico	3	8263	0	0	8266	100.0%	100.0%
	Total							
Enterobacter cloacae	Clinical							
	Contrived							
	in silico	3	8253	0	10	8266	100.0%	99.9%
	Total							
Klebsiella variicola	Clinical							
	Contrived							
	in silico	3	8260	0	3	8266	100.0%	100.0%
	Total							
Klebsiella oxytoca	Clinical							
	Contrived							
	in silico	3	8260	0	3	8266	100.0%	100.0%
	Total							
Morganella morganii	Clinical							
	Contrived							
	in silico	3	8262	0	1	8266	100.0%	100.0%
	Total							
Proteus vulgaris	Clinical							
	Contrived							
	in silico	3	8256	0	7	8266	100.0%	99.9%
	Total							
Providencia rettgeri	Clinical							
	Contrived							
	in silico	3	8260	0	3	8266	100.0%	100.0%
	Total							
Providencia stuartii	Clinical							
	Contrived							
	in silico	3	8260	0	3	8266	100.0%	100.0%
	Total							
Raoultella ornithinolytica	Clinical							
	Contrived							
	in silico	3	8260	0	3	8266	100.0%	100.0%
	Total							
Serratia marcescens	Clinical							
	Contrived							
	in silico	3	8245	0	18	8266	100.0%	99.8%
	Total							
Acinetobacter baumanii	Clinical							
	Contrived							
	in silico	3	8244	0	19	8266	100.0%	99.8%
	Total							

Acinetobacter lwolfii	Clinical							
	Contrived							
	in silico	3	8260	0	3	8266	100.0%	100.0%
	Total							
Pseudomonas aeruginosa	Clinical							
	Contrived							
	in silico	3	8260	0	3	8266	100.0%	100.0%
	Total							
Strenotrophomonas maltophilia	Clinical							
	Contrived							
	in silico	3	8252	0	11	8266	100.0%	99.9%
	Total							
Aerococcus species	Clinical							
	Contrived							
	in silico	21	8242	3	0	8266	87.5%	100.0%
	Total							
Anginosus group Streptococci	Clinical							
	Contrived							
	in silico	5	8261	0	0	8266	100.0%	100.0%
	Total							
Streptococcus agalactiae	Clinical							
	Contrived							
	in silico	3	8263	0	0	8266	100.0%	100.0%
	Total							
Enterococcus faecium	Clinical							
	Contrived							
	in silico	3	8260	0	3	8266	100.0%	100.0%
	Total							
Corynebacterium urealyticum	Clinical							
	Contrived							
	in silico	3	8263	0	0	8266	100.0%	100.0%
	Total							
Staphylococcus epidermidis	Clinical							
	Contrived							
	in silico	3	8263	0	0	8266	100.0%	100.0%
	Total							
Staphylococcus lugdunensis	Clinical							
	Contrived							
	in silico	3	8263	0	0	8266	100.0%	100.0%
	Total							
Staphylococcus saprophyticus	Clinical							
	Contrived							
	in silico	3	8263	0	0	8266	100.0%	100.0%
	Total							
Mitis group Streptococci	Clinical							
	Contrived							
	in silico	3	8262	0	1	8266	100.0%	100.0%
	Total							
Anaerococcus vaginalis	Clinical							
	Contrived							
	in silico	3	8263	0	0	8266	100.0%	100.0%
	Total							
Bacteroides fragilis	Clinical							
	Contrived							
	in silico	3	8263	0	0	8266	100.0%	100.0%
	Total							
Prevotella species	Clinical							
	Contrived							
	in silico	9	8257	0	0	8266	100.0%	100.0%
	Total							
Gardnerella vaginalis	Clinical							
	Contrived							
	in silico	3	8263	0	0	8266	100.0%	100.0%
	Total							
Negative Organisms*	Contrived							
	in silico	7977	289	0	0	8266	100.0%	100.0%
	Total							

Supplementary Table 3B. Performance Characteristics of in silico Studies per Key Fungal Urogenital Taxa

Key pathogen	Method	TP	TN	FN	FP	Total	Sensitivity	Specificity
Candida albicans	Clinical							
	Contrived							
	in silico	9	4133	0	0	4142	100.0%	100.0%
	Total							
Candida auris	Clinical							
	Contrived							
	in silico	9	4133	0	0	4142	100.0%	100.0%
	Total							
Candida dubliniensis	Clinical							
	Contrived							
	in silico	9	4133	0	0	4142	100.0%	100.0%
	Total							
Candida glabrata	Clinical							
	Contrived							
	in silico	9	4133	0	0	4142	100.0%	100.0%
	Total							
Candida guilliermondii	Clinical							
	Contrived							
	in silico	9	4133	0	0	4142	100.0%	100.0%
	Total							
Candida kefyr	Clinical							
	Contrived							
	in silico	9	4133	0	0	4142	100.0%	100.0%
	Total							
Candida krusei	Clinical							
	Contrived							
	in silico	9	4133	0	0	4142	100.0%	100.0%
	Total							
Candida lusitaniae	Clinical							
	Contrived							
	in silico	9	4133	0	0	4142	100.0%	100.0%
	Total							
Candida parapsilopsis	Clinical							
	Contrived							
	in silico	9	4133	0	0	4142	100.0%	100.0%
	Total							
Candida tropicalis	Clinical							
	Contrived							
	in silico	9	4133	0	0	4142	100.0%	100.0%
	Total							
Negative Organisms*	Contrived							
	in silico	4052	90	0	0	4142	100.0%	100.0%
	Total							

Supplementary Table 4. Summary of *in silico* study cross reactive species

Analyte	# of samples	Cross reactive species
Acinetobacter baumanii	19	*Acinetobacter genomosp*
Acinetobacter lwoffii	3	*Acinetobacter pseudolwoffii*
Enterobacter cloacae	10	*Enterobacter chengduensis*
Enterococcus faecium	3	*Enterococcus lactis*
Escherichia coli	48	*Escherichia marmotae*
Klebsiella oxytoca	3	*Klebsiella grimontii*
Klebsiella pneumoniae	13	*Klebsiella quasipneumoniae*
Klebsiella variicola	3	*Klebsiella quasivariicola*
Morganella morganii	1	*Morganella psychrotolerans*
Proteus mirabilis	8	*Proteus penneri*
Proteus vulgaris	7	*Proteus faecis*
Providencia rettgeri	3	*Providencia huaxiensis*
Providencia stuartii	3	*Providencia thailandensis*
Pseudomonas aeruginosa	3	*Pseudomonas paraeruginosa*
Raoultella ornithinolytica	3	*Raoultella planticola*
Serratia marcescens	18	*Serratia nematodiphila*
Staphylococcus aureus	11	*Staphylococcus roterodami*
Stenotrophomonas maltophilia	11	*Stenotrophomonas indicatrix*
Streptococcus mitis	1	*Streptococcus pseudopneumoniae*
**Simulated Reads Species**	**# of samples**	**Cross reactive species**
Proteus vulgaris	2	*Proteus terrae*
Serratia nematodiphila	1	*Serratia ureilytica*
Streptococcus pseudopneumoniae	1	*Streptococcus pneumoniae*
Staphylococcus singaporensis	3	*Staphylococcus haemolyticus*

Supplementary Table 5. Comparator testing was used to resolve discrepancies between the BIOTIA-DX and culture. A combination of qPCR assays and Sanger sequencing were used for resolving such discrepancies. Assays are summarized in Supplementary Table 1.

qPCR Comparator Testing Results

Sample	Analyte	Prediction Probability	Relative abundance	Biotia-ID Result	Comparator result	qPCR Ct value	Outcome
URV-104	*Enterococcus faecalis*	0.988	0.198	Detected	Detected	16.07	True Positive
URV-110	*Enterococcus faecalis*	0.999	0.135	Detected	Detected	15.79	True Positive
URV-118	*Enterococcus faecalis*	0.999	0.215	Detected	Detected	24.00	True Positive
URV-122	*Enterococcus faecalis*	0.972	0.081	Detected	Detected	16.76	True Positive
URV-127	*Enterococcus faecalis*	0.998	0.021	Detected	Detected	21.29	True Positive
URV-130	*Enterococcus faecalis*	0.972	0.179	Detected	Detected	19.99	True Positive
URV-132	*Enterococcus faecalis*	0.999	0.398	Detected	Detected	27.53	True Positive
URV-133	*Enterococcus faecalis*	0.962	0.019	Detected	Detected	25.07	True Positive
URV-134	*Enterococcus faecalis*	0.984	0.008	Detected	Detected	19.91	True Positive
URV-137	*Enterococcus faecalis*	0.999	0.120	Detected	Detected	24.43	True Positive
URV-139	*Enterococcus faecalis*	0.595	0.012	Detected	Not_detected	Undetermined	False Positive
URV-146	*Enterococcus faecalis*	0.999	0.041	Detected	Detected	17.30	True Positive
URV-157	*Enterococcus faecalis*	0.999	0.384	Detected	Detected	16.81	True Positive
URV-163	*Enterococcus faecalis*	0.999	0.739	Detected	Detected	13.77	True Positive
URV-170	*Enterococcus faecalis*	0.999	0.194	Detected	Detected	21.68	True Positive
URV-172	*Enterococcus faecalis*	0.999	0.279	Detected	Detected	18.98	True Positive
URV-176	*Enterococcus faecalis*	0.999	0.090	Detected	Detected	17.53	True Positive
URV-180	*Enterococcus faecalis*	0.999	0.575	Detected	Detected	21.81	True Positive
URV-181	*Enterococcus faecalis*	0.963	0.989	Detected	Detected	14.67	True Positive
URV-182	*Enterococcus faecalis*	0.999	0.155	Detected	Detected	16.58	True Positive
URV-184	*Enterococcus faecalis*	0.676	0.013	Detected	Detected	27.10	True Positive
URV-190	*Enterococcus faecalis*	0.999	0.061	Detected	Detected	19.73	True Positive
URV-220	*Enterococcus faecalis*	0.982	0.805	Detected	Detected	15.03	True Positive
URV-226	*Enterococcus faecalis*	0.999	0.663	Detected	Detected	14.43	True Positive
URV-227	*Enterococcus faecalis*	0.998	0.021	Detected	Detected	26.06	True Positive
URV-232	*Enterococcus faecalis*	0.999	0.305	Detected	Detected	21.34	True Positive
URV-248	*Enterococcus faecalis*	0.999	0.924	Detected	Detected	16.66	True Positive
URV-258	*Enterococcus faecalis*	0.115	0.001	Not detected	Detected	24.59	False Negative
URV-259	*Enterococcus faecalis*	0.402	0.013	Not detected	Detected	24.6	False Negative
URV-261	*Enterococcus faecalis*	0.999	0.895	Detected	Detected	16.26	True Positive
URV-272	*Enterococcus faecalis*	0.999	0.616	Detected	Detected	15.76	True Positive
URV-278	*Enterococcus faecalis*	0.999	0.663	Detected	Detected	16.33	True Positive
URV-280	*Enterococcus faecalis*	0.999	0.046	Detected	Detected	22.42	True Positive
URV-283	*Enterococcus faecalis*	0.999	0.756	Detected	Detected	22.47	True Positive
URV-288	*Enterococcus faecalis*	0.999	0.061	Detected	Detected	24.09	True Positive
URV-291	*Enterococcus faecalis*	0.999	0.133	Detected	Detected	17.96	True Positive
URV-111	*Escherichia coli*	0.999	0.115	Detected	Detected	15.96	True Positive
URV-113	*Escherichia coli*	0.391	0.025	Not detected	Detected	19.96	False Negative
URV-114	*Escherichia coli*	0.998	0.104	Detected	Detected	18.05	True Positive
URV-117	*Escherichia coli*	0.999	0.020	Detected	Detected	18.10	True Positive
URV-130	*Escherichia coli*	0.999	0.133	Detected	Detected	30.40	True Positive
URV-131	*Escherichia coli*	0.999	0.840	Detected	Detected	17.06	True Positive
URV-137	*Escherichia coli*	0.020	0.000	Not detected	Not_detected	Undetermined	True Negative
URV-139	*Escherichia coli*	0.000	0.000	Not detected	Not Detected	Undetermined	True Negative
URV-141	*Escherichia coli*	0.000	0.000	Not detected	Not Detected	Undetermined	True Negative
URV-173	*Escherichia coli*	0.814	0.043	Detected	Detected	19.11	True Positive
URV-178	*Escherichia coli*	0.971	0.098	Detected	Detected	18.50	True Positive
URV-257	*Escherichia coli*	0.999	0.027	Detected	Detected	21.83	True Positive
URV-258	*Escherichia coli*	0.999	0.713	Detected	Detected	11.46	True Positive
URV-268	*Escherichia coli*	0.801	0.041	Detected	Detected	22.08	True Positive
URV-280	*Escherichia coli*	0.997	0.675	Detected	Detected	19.01	True Positive
URV-282	*Escherichia coli*	0.999	0.057	Detected	Detected	26.26	True Positive
URV-289	*Escherichia coli*	0.999	0.677	Detected	Detected	14.22	True Positive

URV-110	*Klebsiella pneumoniae*	0.801	0.012	Detected	Detected	15.81	True Positive
URV-122	*Klebsiella pneumoniae*	0.999	0.159	Detected	Detected	14.59	True Positive
URV-132	*Klebsiella pneumoniae*	0.046	0.000	Detected	Detected	37.81	True Positive
URV-147	*Klebsiella pneumoniae*	0.999	0.071	Detected	Detected	26.79	True Positive
URV-169	*Klebsiella pneumoniae*	0.953	0.043	Detected	Detected	14.82	True Positive
URV-184	*Klebsiella pneumoniae*	0.999	0.140	Detected	Detected	21.88	True Positive
URV-255	*Klebsiella pneumoniae*	0.781	0.002	Detected	Detected	25.35	True Positive
URV-262	*Klebsiella pneumoniae*	0.955	0.003	Detected	Detected	23.69	True Positive
URV-264	*Klebsiella pneumoniae*	0.422	0.004	Not detected	Detected	21.42	False Negative
URV-280	*Klebsiella pneumoniae*	0.801	0.011	Detected	Detected	25.16	True Positive
URV-289	*Klebsiella pneumoniae*	0.813	0.016	Detected	Detected	18.48	True Positive
URV-187	*Proteus mirabilis*	0.895	0.008	Detected	Not Detected	Undetermined	False Positive
URV-192	*Proteus mirabilis*	0.999	0.369	Detected	Detected	25.17	True Positive
URV-228	*Proteus mirabilis*	0.999	0.069	Detected	Detected	21.74	True Positive
URV-231	*Proteus mirabilis*	0.998	0.147	Detected	Detected	31.71	True Positive
URV-268	*Proteus mirabilis*	0.998	0.075	Detected	Detected	34.07	True Positive
URV-291	*Staphylococcus aureus*	0.978	0.109	Detected	Not_detected	Undetermined	False Positive
URV-118	*Aerococcus urinae*	0.968	0.001	Detected	Detected	28.17	True Positive
URV-130	*Aerococcus urinae*	0.662	0.001	Detected	Detected	23.52	True Positive
URV-139	*Aerococcus urinae*	0.594	0.001	Detected	Detected	30.94	True Positive
URV-187	*Aerococcus urinae*	0.986	0.005	Detected	Detected	26.56	True Positive
URV-284	*Aerococcus urinae*	0.986	0.002	Detected	Detected	26.70	True Positive
URV-285	*Aerococcus urinae*	0.802	0.001	Detected	Detected	23.11	True Positive
URV-287	*Aerococcus urinae*	0.939	0.002	Detected	Detected	24.04	True Positive
URV-291	*Aerococcus urinae*	0.967	0.001	Detected	Detected	19.91	True Positive
URV-108	*Gardnerella vaginalis*	0.684	0.002	Detected	Not Detected	Undetermined	False Positive
URV-114	*Gardnerella vaginalis*	0.930	0.028	Detected	Detected	18.83	True Positive
URV-125	*Gardnerella vaginalis*	0.948	0.142	Detected	Detected	36.87	True Positive
URV-128	*Gardnerella vaginalis*	0.696	0.029	Detected	Not Detected	Undetermined	False Positive
URV-136	*Gardnerella vaginalis*	0.859	0.003	Detected	Detected	26.86	True Positive
URV-140	*Gardnerella vaginalis*	0.934	0.084	Detected	Detected	22.10	True Positive
URV-163	*Gardnerella vaginalis*	0.875	0.030	Detected	Not Detected	Undetermined	False Positive
URV-167	*Gardnerella vaginalis*	0.964	0.004	Detected	23.927	23.927	True Positive
URV-175	*Gardnerella vaginalis*	0.658	0.084	Detected	Not Detected	Undetermined	False Positive
URV-181	*Gardnerella vaginalis*	0.803	0.002	Detected	21.28	21.28	True Positive
URV-191	*Gardnerella vaginalis*	0.738	0.098	Detected	17.23	17.23	True Positive
URV-223	*Gardnerella vaginalis*	0.970	0.013	Detected	31.97	31.97	True Positive
URV-233	*Gardnerella vaginalis*	0.944	0.083	Detected	15.60	15.60	True Positive
URV-256	*Gardnerella vaginalis*	0.840	0.545	Detected	20.08	20.08	True Positive
URV-263	*Gardnerella vaginalis*	0.994	0.011	Detected	28.93	28.93	True Positive
URV-278	*Gardnerella vaginalis*	0.638	0.031	Detected	Not Detected	Undetermined	False Positive
URV-286	*Gardnerella vaginalis*	0.608	0.178	Detected	Not Detected	Undetermined	False Positive
URV-104	*Prevotella species*	0.986	0.001	Detected	Detected	14.83	True Positive
URV-112	*Prevotella species*	0.998	0.012	Detected	Detected	15.96	True Positive
URV-163	*Prevotella species*	0.874	0.001	Detected	Detected	15.49	True Positive
URV-249	*Prevotella species*	0.998	0.029	Detected	Detected	16.78	True Positive
URV-259	*Prevotella species*	0.992	0.006	Detected	Detected	17.4	True Positive
URV-270	*Prevotella species*	0.994	0.005	Detected	Detected	17.79	True Positive
URV-271	*Prevotella species*	0.961	0.003	Detected	Detected	17.87	True Positive

Sanger Sequencing Comparator Results

Sample	Analyte	Prediction Probability	Relative abundance	Biotia-ID Result	Comparator result	% Identity	Comparator Agreement
URV-146	*Enterococcus faecium*	0.998	0.017	Detected	Detected	100%	True Positive
URV-225	*Enterococcus faecium*	0.961	0.624	Detected	Detected	100%	True Positive
URV-176	*Morganella morganii*	0.988	0.004	Detected	Detected	100%	True Positive
URV-272	*Morganella morganii*	0.998	0.013	Detected	Detected	100%	True Positive
URV-112	*aphylococcus epidermidi*	0.828	0.001	Detected	Detected	100%	True Positive
URV-116	*aphylococcus epidermidi*	0.984	0.005	Detected	Detected	99.50%	True Positive

URV-291	*phylococcus saprophytic*	0.976	0.008	Detected	Detected	99.25%	True Positive
URV-146	*Streptococcus agalactiae*	0.999	0.122	Detected	Detected	100%	True Positive
URV-167	*Streptococcus agalactiae*	0.949	0.004	Detected	Detected	100%	True Positive
URV-186	*Streptococcus agalactiae*	0.930	0.003	Detected	Detected	100%	True Positive
URV-232	*Streptococcus agalactiae*	0.985	0.006	Detected	Detected	100%	True Positive
URV-291	ginosus group Streptoco	0.565	0.002	Detected	Detected	100%	True Positive
URV-140	ginosus group Streptoco	0.827	0.017	Detected	Not Detected	NA	False Positive
URV-275	ginosus group Streptoco	0.515	0.002	Detected	Not Detected	NA	False Positive
URV-287	ginosus group Streptoco	0.538	0.004	Detected	Not Detected	NA	False Positive
URV-154	*rynebacterium urealyticu*	0.998	0.024	Detected	Detected	100%	True Positive
URV-227	*rynebacterium urealyticu*	0.963	0.002	Detected	Detected	100%	True Positive
URV-287	*rynebacterium urealyticu*	0.549	0.000	Detected	Detected	100%	True Positive
URV-288	*rynebacterium urealyticu*	0.645	0.001	Detected	Detected	100%	True Positive
URV-225	*Candida albicans*	0.980	0.042	Detected	Detected	100%	True Positive
URV-230	*Candida albicans*	0.980	0.314	Detected	Detected	100%	True Positive
URV-233	*Candida albicans*	0.986	0.087	Detected	Detected	99.8%	True Positive
URV-260	*Candida albicans*	0.943	0.019	Detected	Detected	100%	True Positive
URV-278	*Candida albicans*	0.996	0.081	Detected	Detected	97.50%	True Positive
SBU-103	*Candida albicans*	0.998	0.171	Detected	Detected	99.8%	True Positive
SBU-161	*Candida albicans*	0.598	0.017	Detected	Detected	99.8%	True Positive
SBU-176	*Candida albicans*	0.963	0.378	Detected	Detected	100.0%	True Positive
URV-148	*Candida glabrata*	0.982	0.679	Detected	Detected	99.9%	True Positive
URV-182	*Candida glabrata*	0.982	0.314	Detected	Detected	98.70%	True Positive
URV-262	*Candida glabrata*	0.982	0.661	Detected	Detected	99.9%	True Positive
URV-288	*Candida glabrata*	0.991	0.047	Detected	Detected	99.90%	True Positive
SBU-021	*Candida glabrata*	0.974	0.347	Detected	Detected	100.00%	True Positive
SBU-047	*Candida glabrata*	0.972	0.305	Detected	Detected	99.80%	True Positive
SBU-137	*Candida glabrata*	0.967	0.016	Detected	Detected	99.88%	True Positive
SBU-162	*Candida glabrata*	0.981	0.075	Detected	Detected	99.76%	True Positive
SBU-017	*Candida tropicalis*	0.733	0.108	Detected	Detected	99.80%	True Positive
SBU-018	*Candida tropicalis*	0.988	0.392	Detected	Detected	100.00%	True Positive
SBU-027	*Candida tropicalis*	0.977	0.269	Detected	Detected	99.80%	True Positive
SBU-074	*Candida tropicalis*	0.577	0.171	Detected	Detected	98.80%	True Positive
SBU-017	*Candida krusei*	0.696	0.058	Detected	Detected	100%	True Positive

Supplementary Table 6A. Performance Characteristics fo Key Bacterial Urogenital Taxa Reported with BIOTIA-ID

Key pathogen	Method	TP	TN	FN	FP	Total	Sensitivity	Specificity
Escherichia coli	Clinical	39	204	1	1	245	97.50%	99.51%
	Contrived	157	1096	0	13	1266	100.00%	98.83%
	in silico	3	8239	0	24	8266	100.00%	99.71%
	Total	199	9539	1	38	9777	99.50%	99.60%
Klebsiella pneumoniae	Clinical	28	214	2	1	245	93.33%	99.53%
	Contrived	166	1099	0	1	1266	100.00%	99.91%
	in silico	3	8250	0	13	8266	100.00%	99.84%
	Total	197	9563	2	15	9777	98.99%	99.84%
Proteus mirabilis	Clinical	22	222	0	1	245	100.00%	99.55%
	Contrived	163	1103	0	0	1266	100.00%	100.00%
	in silico	3	8255	0	8	8266	100.00%	99.90%
	Total	188	9580	0	9	9777	100.00%	99.91%
Enterococcus faecalis	Clinical	61	181	2	1	245	96.83%	99.45%
	Contrived	147	1119	0	0	1266	100.00%	100.00%
	in silico	3	8263	0	0	8266	100.00%	100.00%
	Total	211	9563	2	1	9777	99.06%	99.99%
Staphylococcus aureus	Clinical	2	242	0	1	245	100.00%	99.59%
	Contrived	172	1094	0	0	1266	100.00%	100.00%
	in silico	3	8252	0	11	8266	100.00%	99.87%
	Total	177	9588	0	12	9777	100.00%	99.88%
Citrobacter species	Clinical	0	243	0	2	245	-	99.18%
	Contrived	2	1264	0	0	1266	100.00%	100.00%
	in silico	20	8246	0	0	8266	100.0%	100.0%
	Total	22	9753	0	2	9777	100.00%	99.98%
Enterobacter aerogenes	Clinical	0	244	1	0	245	0.00%	100.00%
	Contrived	1	1265	0	0	1266	100.00%	100.00%
	in silico	3	8263	0	0	8266	100.00%	100.00%
	Total	4	9772	1	0	9777	80.00%	100.00%
Enterobacter cloacae	Clinical	0	245	0	0	245	-	100.00%
	Contrived	1	1265	0	0	1266	100.00%	100.00%
	in silico	3	8253	0	10	8266	100.00%	99.88%
	Total	4	9763	0	10	9777	100.00%	99.90%
Klebsiella oxytoca	Clinical	0	244	0	1	245	-	99.59%
	Contrived	1	1265	0	0	1266	100.00%	100.00%
	in silico	3	8260	0	3	8266	100.00%	99.96%
	Total	4	9769	0	4	9777	100.00%	99.96%
Klebsiella variicola	Clinical	1	242	0	2	245	100.00%	99.18%
	Contrived	1	1265	0	0	1266	100.00%	100.00%
	in silico	3	8260	0	3	8266	100.00%	99.96%
	Total	5	9767	0	5	9777	100.00%	99.95%
Morganella morganii	Clinical	2	243	0	0	245	100.00%	100.00%
	Contrived	2	1264	0	0	1266	100.00%	100.00%
	in silico	3	8262	0	1	8266	100.00%	99.99%
	Total	7	9769	0	1	9777	100.00%	99.99%
Proteus vulgaris	Clinical	0	244	1	0	245	0.00%	100.00%
	Contrived	1	1265	0	0	1266	100.00%	100.00%
	in silico	3	8256	0	7	8266	100.00%	99.92%
	Total	4	9765	1	7	9777	80.00%	99.93%
Providencia rettgeri	Clinical	0	244	0	1	245	-	99.59%
	Contrived	1	1265	0	0	1266	100.00%	100.00%
	in silico	3	8260	0	3	8266	100.00%	99.96%
	Total	4	9769	0	4	9777	100.00%	99.96%
Providencia stuartii	Clinical	0	244	0	1	245	-	99.59%
	Contrived	1	1265	0	0	1266	100.00%	100.00%
	in silico	3	8260	0	3	8266	100.00%	99.96%
	Total	4	9769	0	4	9777	100.00%	99.96%
Raoultella ornithinolytica	Clinical	0	245	0	0	245	-	100.00%
	Contrived	1	1265	0	0	1266	100.00%	100.00%
	in silico	3	8260	0	3	8266	100.00%	99.96%
	Total	4	9770	0	3	9777	100.00%	99.97%
Serratia marcescens	Clinical	0	245	0	0	245	-	100.00%
	Contrived	1	1265	0	0	1266	100.00%	100.00%
	in silico	3	8245	0	18	8266	100.00%	99.78%
	Total	4	9755	0	18	9777	100.00%	99.82%
Acinetobacter baumanii	Clinical	0	244	0	1	245	-	99.59%
	Contrived	4	1262	0	0	1266	100.00%	100.00%
	in silico	3	8244	0	19	8266	100.00%	99.77%
	Total	7	9750	0	20	9777	100.00%	99.80%
	Clinical	0	245	0	0	245	-	100.00%

Acinetobacter lwolfii	Contrived	1	1265	0	0	1266	100.00%	100.00%
	in silico	3	8260	0	3	8266	100.0%	100.0%
	Total	4	9770	0	3	9777	100.00%	99.97%
Pseudomonas aeruginosa	Clinical	0	244	0	1	245	-	100%
	Contrived	7	1259	0	0	1266	100.00%	100.00%
	in silico	3	8260	0	3	8266	100.0%	100.0%
	Total	10	9763	0	4	9777	100.00%	99.96%
Strenotrophomonas maltophilia	Clinical	0	244	0	1	245	-	100%
	Contrived	1	1265	0	0	1266	100.00%	100.00%
	in silico	3	8252	0	11	8266	100.0%	100.0%
	Total	4	9761	0	12	9777	100.00%	99.88%
Aerococcus species	Clinical	8	237	0	0	245	100.00%	100.00%
	Contrived	2	1264	0	0	1266	100.00%	100.00%
	in silico	21	8242	3	0	8266	87.50%	100.00%
	Total	31	9743	3	0	9777	91.18%	100.00%
Anginosus group Streptococci	Clinical	1	235	0	9	245	100.00%	96.31%
	Contrived	1	1265	0	0	1266	100.00%	100.00%
	in silico	5	8261	0	0	8266	100.00%	100.00%
	Total	7	9761	0	9	9777	100.00%	99.91%
Corynebacterium urealyticum	Clinical	4	241	0	0	245	100.00%	100.00%
	Contrived	1	1265	0	0	1266	100.00%	100.00%
	in silico	3	8263	0	0	8266	100.00%	100.00%
	Total	8	9769	0	0	9777	100.00%	100.00%
Enterococcus faecium	Clinical	2	241	0	2	245	100.00%	99.18%
	Contrived	1	1003	0	0	1004	100.00%	100.00%
	in silico	3	8260	0	3	8266	100.00%	99.96%
	Total	6	9504	0	5	9515	100.00%	99.95%
Mitis group Streptococci	Clinical	0	245	0	0	245	-	100.00%
	Contrived	1	1265	0	0	1266	100.00%	100.00%
	in silico	3	8262	0	1	8266	100.00%	99.99%
	Total	4	9772	0	1	9777	100.00%	99.99%
Staphylococcus epidermidis	Clinical	2	242	0	1	245	100.00%	99.59%
	Contrived	5	1261	0	0	1266	100.00%	100.00%
	in silico	3	8263	0	0	8266	100.00%	100.00%
	Total	10	9766	0	1	9777	100.00%	99.99%
Staphylococcus lugdunensis	Clinical	0	245	0	0	245	-	100.00%
	Contrived	1	1265	0	0	1266	100.00%	100.00%
	in silico	3	8263	0	0	8266	100.00%	100.00%
	Total	4	9773	0	0	9777	100.00%	100.00%
Staphylococcus saprophyticus	Clinical	1	242	0	2	245	100.00%	99.18%
	Contrived	1	1265	0	0	1266	100.00%	100.00%
	in silico	3	8263	0	0	8266	100.00%	100.00%
	Total	5	9770	0	2	9777	100.00%	99.98%
Other Staphylococci	Clinical	0	245	0	0	245	-	100.00%
	Contrived	9	1257	0	0	1266	100.00%	100.00%
	in silico	3	8263	0	0	8266	100.00%	100.00%
	Total	12	9765	0	0	9777	100.00%	100.00%
Streptococcus agalactiae	Clinical	4	241	0	0	245	100.00%	100.00%
	Contrived	1	1265	0	0	1266	100.00%	100.00%
	in silico	3	8263	0	0	8266	100.00%	100.00%
	Total	8	9769	0	0	9777	100.00%	100.00%
Anaerococcus vaginalis	Clinical	0	245	0	0	245	-	100.00%
	Contrived	1	1265	0	0	1266	100.00%	100.00%
	in silico	3	8263	0	0	8266	100.00%	100.00%
	Total	4	9773	0	0	9777	100.00%	100.00%
Bacteroides fragilis	Clinical	0	245	0	0	245	-	100.00%
	Contrived	7	1259	0	0	1266	100.00%	100.00%
	in silico	3	8263	0	0	8266	100.00%	100.00%
	Total	10	9767	0	0	9777	100.00%	100.00%
Prevotella species	Clinical	7	238	0	0	245	100.00%	100.00%
	Contrived	7	1259	0	0	1266	100.00%	100.00%
	in silico	9	8257	0	0	8266	100.00%	100.00%
	Total	23	9754	0	0	9777	100.00%	100.00%
Gardnerella vaginalis	Clinical	11	228	0	6	245	100.00%	97.44%
	Contrived	7	1259	0	0	1266	100.00%	100.00%
	in silico	3	8263	0	0	8266	100.00%	100.00%
	Total	21	9750	0	6	9777	100.00%	99.94%
Negative Organisms	Clinical	15	230	0	0	245	100.00%	100.00%
	Contrived	223	1230	0	0	1266	100.00%	100.00%
	in silico	7977	289	0	0	8266	100.00%	100.00%
	Total	8215	1749	0	0	9777	100.00%	100.00%

Supplementary Table 6B. Performance Characteristics fo Key Fungal Urogenital Taxa Reported with BIOTIA-ID

Key pathogen	Method	TP	TN	FN	FP	Total	Sensitivity	Specificity
Candida albicans	Clinical	8	28	0	0	36	100.0%	100.0%
	Contrived	132	795	1	0	928	99.2%	100.0%
	in silico	9	4133	0	0	4142	100.0%	100.0%
	Total	149	4956	1	0	5106	99.3%	100.0%
Candida auris	Clinical	0	36	0	0	36	-	100.0%
	Contrived	129	797	0	2	928	100.0%	99.7%
	in silico	9	4133	0	0	4142	100.0%	100.0%
	Total	138	4966	0	2	5106	100.0%	100.0%
Candida dubliniensis	Clinical	0	36	0	0	36	-	100.0%
	Contrived	10	914	0	0	924	100.0%	100.0%
	in silico	9	4133	0	0	4142	100.0%	100.0%
	Total	19	5083	0	0	5102	100.0%	100.0%
Candida glabrata	Clinical	8	28	0	0	36	100.0%	100.0%
	Contrived	135	793	0	0	928	100.0%	100.0%
	in silico	9	4133	0	0	4142	100.0%	100.0%
	Total	152	4954	0	0	5106	100.0%	100.0%
Candida guilliermondii	Clinical	0	36	0	0	36	-	100.0%
	Contrived	10	918	0	0	928	100.0%	100.0%
	in silico	9	4133	0	0	4142	100.0%	100.0%
	Total	19	5087	0	0	5106	100.0%	100.0%
Candida kefyr	Clinical	0	36	0	0	36	-	100.0%
	Contrived	10	918	0	0	928	100.0%	100.0%
	in silico	9	4133	0	0	4142	100.0%	100.0%
	Total	19	5087	0	0	5106	100.0%	100.0%
Candida krusei	Clinical	1	35	0	0	36	-	100.0%
	Contrived	137	791	0	0	928	100.0%	100.0%
	in silico	9	4133	0	0	4142	100.0%	100.0%
	Total	147	4959	0	0	5106	100.0%	100.0%
Candida lusitaniae	Clinical	0	36	0	0	36	-	100.0%
	Contrived	10	882	0	0	928	100.0%	100.0%
	in silico	9	4133	0	0	4142	100.0%	100.0%
	Total	19	5051	0	0	5106	100.0%	100.0%
Candida parapsilosis	Clinical	0	36	0	0	36	-	100.0%
	Contrived	139	789	0	0	928	100.0%	100.0%
	in silico	9	4133	0	0	4142	100.0%	100.0%
	Total	148	4958	0	0	5106	100.0%	100.0%
Candida tropicalis	Clinical	4	32	0	0	36	100.0%	100.0%
	Contrived	134	794	0	0	928	100.0%	100.0%
	in silico	9	4133	0	0	4142	100.0%	100.0%
	Total	147	4959	0	0	5106	100.0%	100.0%
Negative Organisms	Clinical	9	191	0	0	200	100.0%	100.0%
	Contrived	79	849	0	0	928	100.0%	100.0%
	in silico	4052	90	0	0	4142	100.0%	100.0%
	Total	4140	1130	0	0	5270	100.0%	100.0%
